# A *“*CPApoptosis*”* nano-actuator switches immune-off solid tumors to immune-on for fueling T-cell- based immunotherapy

**DOI:** 10.7150/thno.105867

**Published:** 2025-03-03

**Authors:** Ying Luo, Yi Wang, Bo Liu, Yun Liu, Wenli Zhang, Sijin Chen, Xiyue Rong, Lian Xu, Qianying Du, Jia Liu, Jie Xu, Haitao Ran, Zhigang Wang, Dajing Guo

**Affiliations:** 1 Department of Radiology, Second Affiliated Hospital of Chongqing Medical University, Chongqing, 400010, PR China.; 2 Chongqing Key Laboratory of Ultrasound Molecular Imaging & Department of Ultrasound, Second Affiliated Hospital of Chongqing Medical University, Chongqing, 400010, PR China.

**Keywords:** cuproptosis, pyroptosis, apoptosis, immunogenic cell death, bacterial outer membrane vesicle

## Abstract

**Background:** Most anticancer agents induce tumor apoptosis, but they often lack immunogenicity and display limited success when combined with mainstream immunotherapies, thus killing cancer cells through multiple cell death modalities as well as switching immune-off tumors to immune-on is a strategy with great promise. To this end, we developed a CPApoptosis (cuproptosis, pyroptosis, apoptosis) nano-actuator for immunologically cold solid tumors.

**Methods:** In this study, elesclomol (ES), a mitochondrial targeting copper transporter, was encapsulated within bacterial outer membrane vesicles (OMVs). These OMVs were then surface-modified via metal-phenolic self-assembly using Cu^2+^ and tannic acid (TA).

**Results:** The Cu^2+^ and ES were released from the OMVs in a pH-dependent manner. OMV activated the non-canonical pyroptotic pathway, leading to cell membrane rupture. Cu^2+^ on the one hand was transported to the mitochondria for cuproptosis facilitated by ES, on the other hand, Cu^2+^ was reduced into Cu^+^ by TA, which catalyzed ROS production to induce oxidative apoptosis. Simultaneously, TA degraded glutathione (GSH), sensitizing cells to cuproptosis. The multifactorial cell death mechanisms led to the release of immunogenic factors from lysed tumor cells, stimulating dendritic cell maturation and recruiting cytotoxic T cells. This immune response was further amplified by αPD-L1 antibody treatment.

**Conclusion:** The CPApoptosis nano-actuator represents a promising approach to enhance current cancer therapies, inducing both tumor cell death and a robust immune response, with the potential for long-lasting protective effects.

## Introduction

Most tumors remain difficult to treat due to their marked propensity for metastasis and relapse driven by intra-tumoral heterogeneity and tumor immune evasion 1. A promising approach to prevent tumor metastasis and relapse is to induce immunogenic cell death (ICD) that simultaneously kills cancer cells and generates antitumor immune memory by evoking the dormant immune systems, which in turn determines the long-term success of anticancer therapies 2. In clinical settings, most anticancer drugs rely on apoptosis, a form of programmed cell death that the body utilizes to decompose over a billion cells each day without inducing an immunostimulatory response, leaving the immune system in an “off” state 3. While some chemotherapeutic drugs can induce excessive intracellular reactive oxygen species (ROS), leading to calreticulin (CRT) translocation and making dying tumor cells immunogenic, the release of damage-associated molecular patterns (DAMPs) and tumor-associated antigens (TAAs) into the extracellular space remains insufficient to trigger a full immune response 4. Therefore, it is imperative to develop a strategy to elicit extensive tumor cell damage while simultaneously eliciting strong immunogenicity and adjuvanticity. Beyond apoptosis, numerous studies have explored alternative forms of cell death, such as pyroptosis, ferroptosis, cuproptosis, and integrated cell death (PANoptosis), that are associated with enhanced immunogenicity 5. Cancer cells are known to evade single forms of cell death, which contributes to treatment resistance. Therefore, combining multiple cell death modalities holds great potential for more effective cancer therapies, such as PANoptosis. However, few studies have examined the spatial and temporal regulation of multiple cell death pathways, with PANoptosis being the most studied in the context of bacterial infections and mediated by PANoptosomes 6-11.

In contrast to immunologically silent apoptosis, pyroptosis is a highly immunogenic form of cell death characterized by cell swelling and rupture, releasing DAMPs, TAAs, and inflammatory factors that enhance immune activation 12. Pyroptosis is triggered by the cleavage of gasdermin (GSDM) proteins by inflammatory caspases, releasing the gasdermin-N (GSDM-N) domain, which can perforate the plasma membrane and increase immunogenicity. It has been shown that bacterial outer membrane vesicles (OMVs) can activate non-canonical pyroptosis via LPS-activated murine caspase-11 or humane caspase-4-dependent GSDMD pathways, as OMVs from Gram-negative bacteria contain abundant LPS 13, 14. Unlike the canonical pathway triggered by the activation of inflammasomes and caspases, the non-canonical caspase-11 pathway is activated by Gram-negative bacteria infection, which is beneficial for mobilizing numerous innate immune cells (macrophages, dendritic cells, natural killer cells, etc.) to combat tumors. Moreover, OMVs have been shown to deliver both hydrophobic and hydrophilic molecules for cancer chemotherapy and transport metal ions for metalloimmunotherapy 15-19. Given the versatility of OMVs, we hypothesized that they could be optimized as an ideal platform for inducing multiple modes of tumor cell death with strong immunogenicity, as the pyroptosis they induce can not only activate immune responses but also facilitate the release of intracellular immunogenic factors.

Cuproptosis, a newly discovered form of cell death, has been shown to antagonize various tumor cell lines and trigger an immune response through copper-based therapies 20. Mechanistically, excessive copper ions induced proteotoxic cell death through the aggregation of dihydrolipoamide acetyltransferase (DLAT), and degradation of iron-sulfur (Fe-S) cluster proteins 21. Recent studies have demonstrated that cuproptosis is highly immunogenic, leading to the production of abundant TAAs and DAMPs, and resulting in dendritic cell (DC) maturation and T-cell activation, which are key components of adaptive antitumor immunity 7, 22-28. However, the discovery of cuproptosis was still in its infancy with no convincing evidence of cell membrane rupture which is important for the outflow of immunogenic factors. As a prerequisite of efficient cuproptosis, an important technological difficulty is to spatiotemporally control Cu ion release in tumor cells, since the short circulation lifetime, dose-limiting systemic toxicity, and poor selectivity of Cu ion toward tumors make it unavailable to achieve single-ion delivery. Additionally, the intracellular glutathione (GSH) content and the lack of Cu²⁺ transporters can limit the efficacy of cuproptosis 26, 28. To maximize the antitumor efficacy of cuproptosis, it is essential to combine GSH-consuming agents, copper ion transporters (e.g., Elesclomol, ES), and other forms of cell death that can induce membrane rupture. In this context, Yang and co-workers developed novel drug delivery systems based on CuO_2_ for amplified cuproptosis by effectively regulating the tumor acidity, GSH levels, and oxygen-depleted tumor microenvironment 29, 30.

Building on the fact that OMVs can induce non-canonical pyroptosis and serve as effective drug delivery vehicles due to their modifiability and biodegradability, we hypothesized that OMVs could be engineered to trigger both pyroptosis and cuproptosis simultaneously 31, 32. ES, a copper ion transporter, selectively transports Cu²⁺ into mitochondria, leading to continuous copper accumulation 33. To address this, natural polyphenols like tannic acid (TA) were employed, as they exhibit good biocompatibility and can form stable metal-phenolic networks (MPNs) on the OMV surface by coordinating with metal ions like Cu²⁺ 34, 35. Building on previous studies that used ferric ions (Fe³⁺) and TA to form MPNs on OMVs 36, we hypothesized that Cu^2+^ and TA could form a stable coordination bond on the surface of OMV due to its strong chelating ability, preventing early recognition by the mononuclear phagocytic system. Furthermore, under acidic conditions or in the presence of reducing agents, TA could degrade GSH through redox reactions, thereby sensitizing cells to cuproptosis 36-40. After dissociation from the OMV in the acidic intracellular environment, partial Cu²⁺ could be reduced to Cu⁺, which then catalyzes ROS production via Fenton-like reactions, leading to oxidative stress and apoptosis.

**Scheme 1:**
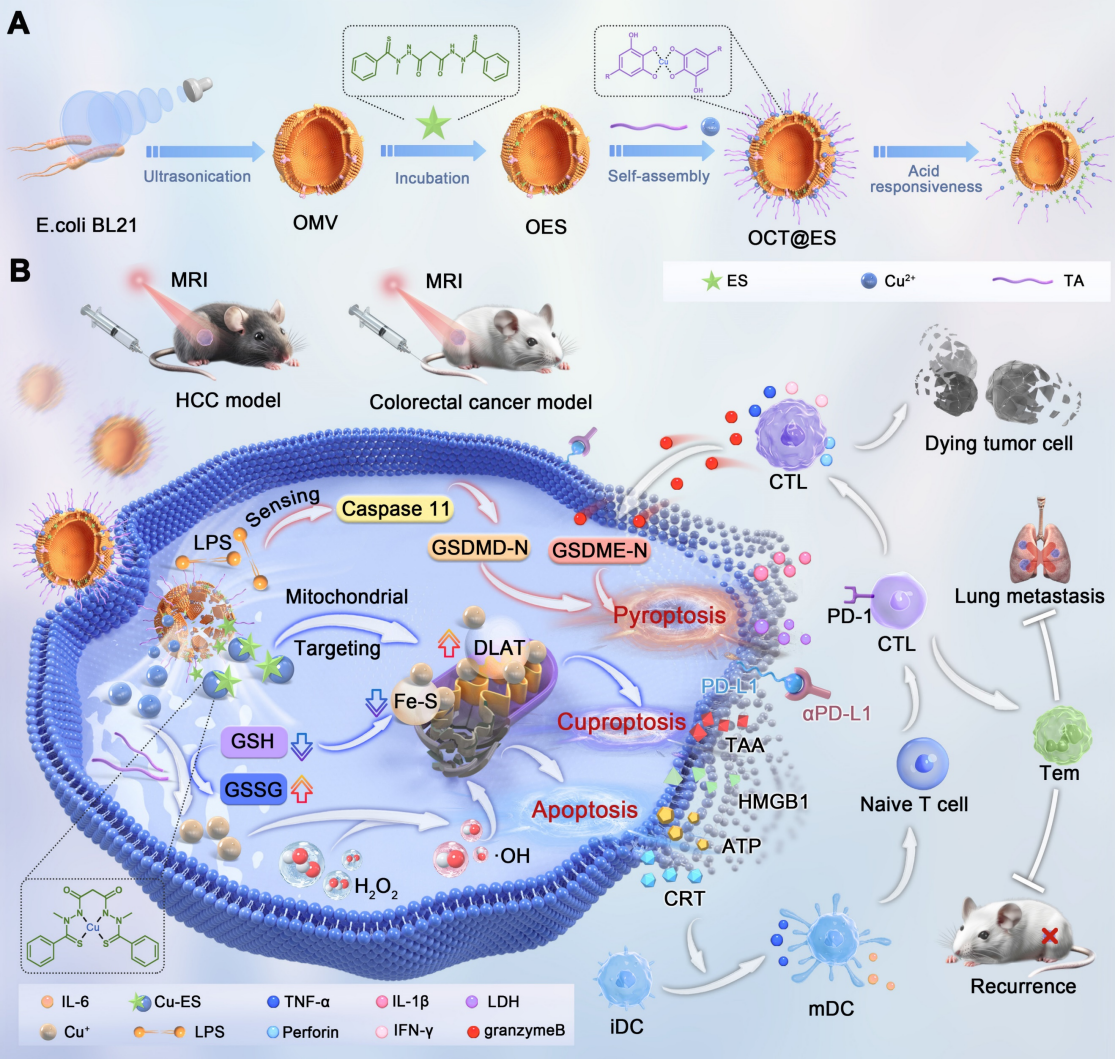
(A) The synthetic process of the OCT@ES. (B) The illustrative mechanism of OCT@ES-induced ternary cell death modalities (cuproptosis, pyroptosis, and apoptosis) accompanied by antitumor immune activation locally and systematically for long-term protection.

In this study, we engineered a CPApoptosis (cuproptosis, pyroptosis, and apoptosis) nano-actuator to simultaneously induce tumor cell death and convert immune-silent tumors to immune-active ones **(Scheme 1)**. By design, OMV was extracted from *E. coli* bacteria via sonification for mass production. Then, the hydrophobic drug, ES, was loaded into the interspace of the OMV lipid bilayer via diffusion, followed by coordination-mediated interfacial interaction between Cu^2+^ and TA for the assembly of Cu-TA on the OMV surface 16, 17, 19, resulting in the product designated as OCT@ES. Mechanistically speaking, OCT@ES passively targeted the tumor site via an enhanced permeability retention (EPR) effect, followed by tumor cell endocytosis. In the acidic intracellular environment of the lysosome, Cu-TA dissociated, releasing Cu²⁺ and TA. TA acted as a reducing agent to degrade GSH, sensitizing the cells to cuproptosis. Cu²⁺ could be reduced to Cu⁺, triggering ROS production and apoptosis, or bind to ES to form a Cu-ES complex that targeted the mitochondria to initiate cuproptosis. In concert with OMV-induced non-canonical pyroptosis, these three cell death modalities resulted in a strong immunogenic response, as evidenced by the release of ICD factors, including TAAs, CRT, HMGB1, ATP, IL-1β, and LDH. Additionally, we demonstrated that the CPApoptosis nano-actuator, in combination with anti-PD-L1 antibody treatment, elicited robust T-cell-mediated adaptive immunity, as evidenced by effective dendritic cell maturation and cytotoxic T-cell mobilization. Finally, the accumulation of the CPApoptosis nano-actuator at the tumor site was visualized using T_1_-weighted MRI. Overall, this study provided a nano immuno-engineering approach to achieve multi-pathway cell death and flick the immunity switch temporal-spatially for immunologically silent tumors.

## Materials and Methods

### Materials

CuCl_2_·2H_2_O and tannic acid were obtained from Sigma Aldrich (USA). Elesclomol was obtained from GLPBIO company (USA). BeyoPure™ LB Broth (premixed powder), protease inhibitor cocktail, Ethylene Diamine Tetraacetic Acid (EDTA), streptomycin-penicillin, trypsin, DAPI staining agent, CCK-8, and DiI were bought from Beyotime Biotechnology (Jiangsu, China). HEPES-NaOH was bought from a Bio-sharp company. DTNB was acquired from Aladdin (Shanghai, China). The Dulbecco's modified Eagle's medium (DMEM) was purchased from Boster (Wuhan, China). JC-1 dye was obtained from MedChemExpress (USA). 1,1-dioctadecyl-3,3,3,3-tetramethylindo tricarbocyanine iodide (DiR) was bought from AAT Bioquest (CA, USA). Annexin V-FITC/PI Apoptosis Kit was bought from Elascience Biotech nology (Wuhan, China). Calcein-AM/PI Double Staing Kit and ROS assay kit were bought from Dojindo Laboratories (Kumamoto, Japan). Three-color pre-stained protein marker was purchased from Shandong Sparkjade Biotechnology Co., Ltd. Anti-ADX, anti-LIAS, anti-GSDMD, and anti-Caspase-11 antibodies were obtained from Abcam (Britain). Anti-DLAT antibody was purchased from CST (USA). The enzyme-linked immunosorbent assays (ELISAs), including ATP, HMGB1, IL-1β, LDH, TNF-α, and IL-6 were acquired from Bioswamp (Wuhan, China). αPD-L1 antibody was bought from Bio X cell (USA). FITC/PB450 CD11c antibody, PB450 CD11c antibody, PE/APC anti-mouse CD80 antibody, APC/PE anti-mouse CD86 antibody, Violet610/APC CD3 antibody, KO525/PE CD8 antibody, PC5.5 CD4 antibody, PE FOXP3 antibody, FITC granzyme B antibody, PC7 IFNγ antibody, APC PD-1 antibody, APC perforin antibody, PE TNF-α antibody, FITC anti-mouse CD44, PerCP/Cyanine 5.5 anti-mouse CD62L, the transcription factor staining buffer were purchased from Biolegend (San Diego, CA, USA). All the chemicals were used as purchased without further purification.

### Synthesis of OCT@ES

First, OMVs were isolated from *E. coli* BL21 by ultrasonication, following a protocol described in a previous study 41. Briefly, *Escherichia coli* was inoculated into LB medium which was placed in a rotary shaker (180 rpm) at 37 °C overnight. When the OD600 reached 1.2, the culture was centrifuged at 5000 rpm for 10 minutes to collect the bacteria. The bacteria were washed three times with PBS and then suspended in HM lysis buffer (0.25 M sucrose, 1 mM EDTA, 20 mM HEPES-NaOH pH 7.4, and 1× protease inhibitor cocktail). Subsequently, the mixed solution was sonicated by an ultrasonic cell disruptor under an ice bath (power: 30%, time: 10 min). Next, the mixture was centrifuged at 3000 g for 5 min to discard the remaining bacteria and large bacterial debris. The supernatant was further centrifuged at 15,000 × g for 30 minutes to collect the sediment, which was dispersed in cold HM buffer. The suspension was passed through 0.8 μm and 0.45 μm filters at least five times to isolate OMVs. To quantify the OMVs, the solution was lyophilized at -80°C and weighed using a precision balance.

ES (1 mg) was loaded onto OMVs (1 mg/mL suspended in ultrapure water) at 37 ℃ for 2 h on a shaker (300 rpm). Excessive free drugs were removed by ultrafiltration (100 kDa) and washed with PBS three times to obtain OES. By the way, the solution after centrifugation was collected and underwent HPLC to calculate the encapsulation rate and the drug loading efficiency of ES. To assemble Cu-TA onto OES, 70 μL of tannic acid (TA) solution (408 mg/mL in ultrapure water) was added dropwise to 10 mL of OES (1 mg/mL), and the mixture was stirred vigorously for 30 minutes. Then, 70 μL, 140 μL, or 210 μL of CuCl₂·2H₂O (40.8 mg/mL) was added dropwise to the reaction solution to determine the optimal Cu loading capacity. Finally, OCT@ES was obtained by centrifugation (12,000 rpm, 30 minutes, 4°C) and washed with PBS three times. The resulting OCT@ES was lyophilized at -80°C and weighed using a precision balance. A control OCT was prepared similarly without ES loading. For DiI or DiR-labeled OCT@ES, the process was identical, with the addition of DiI or DiR.

### Characterization of OCT@ES

The morphology of OMV and OCT@ES was examined by transmission emission microscopy (TEM) (Tecnai G2 F30 S-TWIN, USA). Elemental mapping of O, S, and Cu was also performed. The hydrodynamic diameters of OMV, OES, OCT, and OCT@ES, as well as the zeta potential of OMV, ES, OES, OCT, and OCT@ES, were analyzed by a surface zeta potential and particle size analyzer (Zetasizer Nano ZS90, Britain). Inductively coupled plasma-mass spectrometry (ICP-MS) (Agilent 7700(MS), USA) was applied to quantify the content of Cu in OCT@ES. X-ray Photoelectron Spectroscopy (XPS) measurement of the valence states of Cu was obtained using the K-Alpha XPS System (Thermo Scientific K-Alpha, USA). X-ray diffraction (XRD) patterns of OMVs and OCT@ES were obtained with a Rigaku Ultima IV X-ray diffractometer (Japan). The hysteresis loop of OCT@ES was measured using a vibrating sample magnetometer (VSM). Fourier transform infrared (FTIR) spectra of OMVs, OES, OCT, OCT@ES, and ES were recorded using a Nicolet iS10 FTIR spectrometer (Thermo Scientific, USA). Protein bands on OMVs and OCT@ES were analyzed by SDS-PAGE. Glutathione peroxidase (GPX)-like activity of OCT@ES was assessed using the 5,5'-Dithiobis-(2-nitrobenzoic acid) (DTNB) solution. Briefly, OCT@ES was suspended in PBS containing 5 mM GSH. After incubation at various time points (0 min, 5 min, 10 min, 15 min, 20 min, 25 min, 30 min, 1 h), the mixture was centrifuged, and the supernatant was collected. DTNB solution (3 mg/mL, 10 µL) was added to the supernatant in a 96-well plate, and absorbance at 412 nm was measured using a microplate reader. To assess pH responsiveness, OCT@ES was dissolved in PBS at pH 5.0, 6.5, and 7.4, and incubated at 37°C with shaking. At various time points (15 min, 30 min, 1 h, 2 h, 4 h, 8 h, 24 h, 48 h, 72 h), 200 µL of the solution was sampled, centrifuged, and analyzed by ICP-MS and HPLC to measure Cu and ES levels.

### Cell culture

Hepa1-6 cells, CT26 cells, and HUVEC cells were purchased from Boster (Wuhan, China). JAWSII cells were obtained from MeisenCTCC (Zhejiang, China). Hepa1-6 cells were cultured in DMEM containing 10% fetal bovine serum (FBS), penicillin (100 U/mL), and streptomycin (100 mg/mL) under a humidified atmosphere of 5% CO_2_ at 37 °C. CT26 cells and HUVEC were cultured in RPMI-1640 medium supplemented with 10% FBS and 1% penicillin-streptomycin at 37 °C with 5% CO_2_. JAWSII cells were cultured in RPMI-1640 medium supplemented with 20% FBS, 1% penicillin- streptomycin, and 5 ng/mL GM-CSF at 37°C with 5% CO_2_. It is worth noting that JAWSII is half adherent and half suspended, while hepa1-6 cells are fully adherent. The culture medium of hepa1-6 cells, CT26 cells, and HUVEC cells were sub-cultured every two days, while the JAWSII cells were sub-cultured every three days.

### Cytotoxicity of OMV, OCT, and OCT@ES

To assess biocompatibility, HUVEC cells were treated with different concentrations of OMV, OCT, and OCT@ES (20, 40, 60, 80, 100 μg/mL) for 24 hours, and cell viability was measured using the CCK-8 assay. For antitumor efficacy, Hepa1-6, CT26, B16, and 4T1 cells were treated similarly, and cell viability was assessed in the same manner.

### The intracellular uptake of DiI-labeled OCT@ES

The cellular uptake of DiI-labeled OCT@ES was explored in hepa1-6 cells under a confocal laser scanning microscope (CLSM) (Nikon, Tokyo, Japan) and flow cytometry (FCM) (FACS Vantage SE, Becton Dickinson, San Jose, CA, USA). For CLSM characterization, hepa1-6 cells (1 × 10^5^ cells per well) were separately cultured in con-focal dishes under a humidified atmosphere of 5% CO_2_ at 37 ℃, followed by cell attachment overnight. Next, the culture medium was discarded and replaced with fresh FBS-free DMEM containing DiI-labeled OCT@ES, followed by co-incubation for 30 min, 1, 2, and 4 h. Then, the confocal dishes were removed from the cell incubator and washed with PBS thrice. Afterward, the cells were fixed with 1 mL of 4% formalin for 15 min. Subsequently, the cells were washed with PBS three times and counter-stained with 100 μL DAPI staining solution for another 15 min. Finally, the cells were washed with PBS and then visualized by CLSM to take fluorescence (FL) images to observe the cellular uptake of DiI-labeled OCT@ES in hepa1-6 cells. FCM characterization was also conducted for quantitative analysis. Hepa1-6 cells (2 × 10^5^ cells per well) were cultured in 6-well plates under a humidified atmosphere of 5% CO_2_ at 37 ℃ respectively. Then, the medium was discarded and replaced with FBS-free DMEM containing DiI-labeled OCT@ES, followed by co-incubation for 30 min, 1, 2, and 4 h. Then, cells were collected by trypsin and suspended in 300 μL PBS solution to undergo FCM analysis.

### Apoptosis induced by OCT@ES* in vitro*

To assess the apoptotic effect of OCT@ES on Hepa1-6 cells, live/dead cell staining and flow cytometry (FCM) were performed to evaluate cell viability following various treatments. Hepa1-6 cells (1 × 10⁵ cells per well) were seeded in a six-well plate and incubated overnight in a humidified atmosphere with 5% CO₂ at 37°C. The medium was then replaced with fresh, FBS-free DMEM containing 300 μg/mL of OMV, OCT, or OCT@ES. After a 24-hour incubation, cells were subjected to live/dead cell staining as per the manufacturer's instructions. Specifically, the working solution was prepared by mixing 10 μL of Calcein-AM and 15 μL of PI in 5 mL PBS. Cells were washed three times with PBS, incubated with 100 μL of the working solution for 15 minutes, and visualized under a fluorescence microscope (Nikon Ti-S, Tokyo, Japan). Meantime, after the same treatment, all the cells were also collected by trypsin and stained by Annexin V-FITC and PI in PBS solution for FCM analysis according to specification. To detect the mechanism of OCT@ES-induced apoptosis, DCFH-DA, and JC-1 dye were used to detect the cellular level of ROS and the state of MMP. Experimentally, hepa1-6 cells (1 × 10^5^ cells per well) were separately cultured in con-focal dishes under a humidified atmosphere of 5% CO_2_ at 37 ℃, followed by cell attachment overnight. Then, the hepa1-6 cells were divided into 4 groups and incubated with PBS, OMV, OCT, and OCT@ES for 24 h respectively. Afterward, all cells were stained with DCFH-DA for 15 min, followed by observation under CLSM and FCM. The cells were also stained with JC-1 dye to observe the change in MMP. All dishes were visualized by CLSM. In addition, cells under different treatments were collected for FCM analysis of MMP. MMP was calculated as the ratio of the JC-1 aggregate/monomer.

### Pyroptosis induced by OCT@ES *in vitro*

To observe pyroptotic morphology, Hepa1-6 cells were seeded into six-well plates and treated with PBS, OMV, OCT, or OCT@ES for 12 hours. Cell morphology was examined using an inverted fluorescence microscope. After treatment, proteins were extracted from the cells, and the expression levels of caspase-11, GSDMD-FL, and GSDMD-N were detected by Western blotting (WB) using the appropriate antibodies (1:1000).

### Cuproptosis induced by OCT@ES *in vitro*

To detect the expression of cuproptosis-related markers, CLSM was applied to examine the expression of DLAT and LIAS. Hepa1-6 cells were first seeded into six-well plates at a density of 1×10^6^ per well for 12 h. Subsequently, the cells were treated with PBS, OMV, OCT, or OCT@ES for 24 h. Subsequently, the cells were fixed with 4% paraformaldehyde and permeabilized by 0.3% Triton-X in PBS at room temperature for 1 h, followed by blocking with 5% BSA solution at room temperature for 1 h. The treated hepa1-6 cells were further incubated with anti-DLAT antibody or anti-LIAS antibody (1:500) in antibody diluent at 4 °C overnight and stained with Goat Anti Mouse IgG (H&L) - Alexa Fluor 488 (1:1000) (EF0007, ShandongSparkjade BiotechnologyCo., Ltd.) for 1 h. Afterward, the treated hepa1-6 cells were washed with PBS three times, and then stained with DAPI for 10 min. Finally, the DLAT and LIAS expression were measured by CLSM. Similarly, a WB experiment was done to detect the expression level of DLAT, LIAS, and FDX1. To observe the mitochondrial morphology, hepa1-6 cells were seeded into 6-well plates at a density of 1×10^6^ per well for 12 h. Afterward, the cells were treated with PBS or OCT@ES for 24 h. Last, the cells were collected and fixed by electron microscope fixative and observed by Bio-TEM.

### ICD induced by OCT@ES *in vitro*

CLSM was employed to evaluate the expression of CRT and HMGB1 in Hepa1-6 cells treated with PBS, OMV, OCT, or OCT@ES for 24 hours. Cells were fixed with 4% paraformaldehyde, permeabilized with 0.3% Triton-X, and blocked with 5% BSA. They were then incubated overnight at 4°C with anti-CRT or anti-HMGB1 antibodies (1:500) and stained with Goat Anti-Mouse IgG (H&L) - Alexa Fluor 488 (1:1000) for 1 hour. After washing, cells were stained with DAPI for 10 minutes, and CRT and HMGB1 expression was observed by CLSM. To study ICD effects on immature dendritic cells (DCs), a co-culture transwell system was established using JAWSII cells (seeded in the bottom chamber) and Hepa1-6 cells (in the upper chamber). After cell attachment overnight, the old culture medium was replaced by DMEM medium containing OMV, OCT, and OCT@ES except for the control group. 24 h after co-incubation, the JAWSII cells were obtained and stained with FITC CD11c antibody, PE anti-mouse CD80 antibody, and APC anti-mouse CD86 antibody following the manufacturer's instruction. Finally, FCM analysis was done to assess the proportion of matured DCs. In addition, the supernatant of the cell culture medium was collected and sent for ELISA to detect the content of ATP, HMGB1, IL-1β, LDH, IL-6, and TNF-α. To detect whether mere OMV, OCT, or OCT@ES could influence DC maturation, JAWSII cells were also incubated with PBS, OMV, and OCT@ES alone for 24 h to detect the proportion of CD11b^+^CD80^+^CD86^+^ cells.

### Establishment of syngeneic HCC mouse models and colorectal cancer mouse models

All animal experiments were approved by the Ethics Committee of Chongqing Medical University and Institutional Animal Care. The permit number for the animal experiments is Research Ethics Review No. 223 (2023). C57BL/6J and BALB/c mice (6-8 weeks, male) were bought from the experimental animal center of Chongqing Medical University. 5 × 10^6^ hepa1-6 cells in 100 μL PBS were injected into the right flanks of 6-8-week-old male C57BL/6J mice (5 mice per group) to develop HCC mouse models, while 2×10^6^ CT26 cells in 100 μL PBS were injected into the right flanks of 6-8-week-old male BALB/c mice (5 mice per group) to establish colorectal cancer mouse models.

### Evaluation of antitumor effect *in vivo*

The schematic diagram of the therapeutic procedure for subcutaneous syngeneic HCC and colorectal mouse models is shown in Figure 4a. Subcutaneous HCC mouse models and colorectal mouse models reached a measurable size 7 days post-tumor inoculation. Then, the tumor-bearing mice were randomly assigned to five groups: saline, OMV, αPD-L1, OCT@ES, and the combination of αPD-L1 and OCT@ES. Treatments were administered on the 1^st^, 4^th^, and 8^th^ days. All mice were weighed and the length and width of tumor volume were measured by Vernier calipers once every other day. The tumor volume (V) was calculated through the following formula: V = (L × W^2^) / 2. The observation for tumor treatment lasted for 14 days. Subsequently, the mice were sacrificed, followed by tumor and major organ extraction. After that, all the above tissues extracted from HCC mouse models were fixed with 4% paraformaldehyde for 24 h and subsequently dehydrated, followed by hematoxylin and eosin (H&E) staining assays. Simultaneously, the tumor tissues were also sent for the immunofluorescent (IF) staining of terminal deoxynucleotidyl transferase dUTP nick end labeling (TUNEL) and proliferating cell nuclear antigen (PCNA) to detect tumor apoptosis and proliferation. For TUNEL staining, the tumor sections were incubated with dUTP (1: 50) for 2 h at 37 ℃, followed by DAPI counterstaining. For PCNA staining, the tumor sections were incubated with anti-PCNA antibody (1:3000) overnight at 4 ℃, followed by co-incubation with HRP-polymer Anti Mouse antibody for 1 h and reaction with TYR-570 for 10 min. Simultaneously, the tumor tissues were also sent for DHE staining to detect the expression of ROS. To be specific, the tumor tissues were first frozen under -80 ℃, and then were sliced into sections. These tumor sections were first stained with DAPI working solution for 3min, followed by co-incubation with dihydroethidium (DHE) staining reagent (1: 400) for 1 h. Finally, after being counter-stained with DAPI working solution and sealed by a fluorescent mounting medium, all the tumor sections were observed by a FL microscopy. The average FL signals of TUNEL, PCNA, and DHE were calculated by ImageJ. In addition, survival curves of C57BL/6J mice were monitored every other day for up to 60 days. The mice were considered dead when the tumor volume reached 1500 mm^3^.

### Mechanistic study of the CPApoptosis *in vivo*

To study the mechanism underlying OCT@ES-induced pyroptosis and cuproptosis, HCC mouse models from each treatment group were sacrificed on day 14. Tumor tissues were extracted for IF staining of pyroptosis-related biomarkers (GSDMD) and cuproptosis-related biomarkers (DLAT, LIAS, and FDX1). The expression of immunogenic cell death (ICD) markers (CRT and HMGB1) was also assessed by IF staining. Tumor sections were incubated overnight at 4°C with the following primary antibodies: anti-GSDMD (1:2000), anti-DLAT (1:3000), anti-LIAS (1:2000), anti-FDX1 (1:2000), anti-CRT (1:1000), and anti-HMGB1 (1:2000). After incubation, the sections were co-incubated with HRP-polymer Anti-Rabbit/Mouse antibody for 1 hour and reacted with TYR-570 for 10 minutes. Finally, sections were counterstained with DAPI and sealed with FL mounting medium before being observed using a fluorescence microscope.

### The evaluation of immune activation *in vivo*

To assess the immunomodulatory effects of various treatments (Saline, OMV, OCT@ES, αPD-L1, OCT@ES + αPD-L1) on the immune system, subcutaneous HCC mouse models were sacrificed on day 14. Tumor tissues, tumor-draining lymph nodes (TDLNs), and spleens were then harvested for FCM analysis. Tissues were cut into small pieces and digested with collagenase A (1 mg/mL), DNAase I (0.5 mg/mL), and hyaluronidase (HAase) (1 mg/mL) at 37°C for 30 minutes. A 40 μm cell strainer was used to filter tumor and TDLN fragments, while 70 μm strainers filtered spleen tissue. The resulting cell suspension was centrifuged at 1500 rpm for 5 minutes, and red blood cells were removed using red blood cell lysis buffer. The suspension was then washed with 1% FBS to stop digestion, followed by centrifugation to obtain purified cells from tumor tissues, TDLNs, and spleens. Cells were stained with the following fluorophore-conjugated antibodies: FITC/PB450 CD11c, PB450 CD11c, PE/APC anti-mouse CD80, APC/PE anti-mouse CD86, Violet610/APC CD3, KO525/PE CD8, PC5.5 CD4, PE FOXP3, FITC granzyme B, PC7 IFNγ, APC PD-1, APC perforin, PE TNF-α, FITC anti-mouse CD44, and PerCP/Cyanine 5.5 anti-mouse CD62L. Granzyme B, IFNγ, perforin, and TNF-α are intracellular markers, requiring permeabilization buffer, while FOXP3 is a nuclear factor, requiring a transcription factor staining buffer as per the manufacturer's instructions. FCM analysis was performed to evaluate the immune cell populations. Simultaneously, tumor tissues were collected for IF staining of GSDME, granzyme B, and FOXP3 to assess immune activation. The procedure for IF staining followed standard protocols. Tumor tissues were also collected for ELISA to measure the intratumoral secretion of ATP, HMGB1, IL-1β, and LDH. Serum from each group was analyzed for IL-6 and TNF-α levels.

### The evaluation of the long-term protective effect on tumor-rechallenged models and lung metastasis models

The subcutaneous HCC mouse models were established by the aforementioned method, followed by the same therapeutic interventions. On day 14, primary tumors in the right flanks of mice were surgically removed, followed by wound stitching and sterilization. On day 30, 5 × 10^6^ hepa1-6 cells were rechallenged into the mice's left flanks of mice. The rechallenged tumor growth curves and weight changes were monitored once every other day from day 36 to day 48. The Length and Width of tumors were measured by Vernier calipers to calculate the tumor volume with the same aforementioned calculation formula. On day 49, all mice were sacrificed and the tumors, TDLNs, and spleens were collected. All tumor samples were weighed and photographed. Single-cell suspension was obtained from the tumor tissue, TDLN, and the spleen according to the aforementioned method. Next, cells from the tumor tissues in different treatment groups were stained with PE anti-mouse CD3 and APC anti-mouse CD8a, FITC anti-mouse CD44, and PerCP/Cyanine 5.5 anti-mouse CD62L according to the manufacturer's instructions. Finally, FCM was conducted to analyze the ratio of effector Tem cells in each tissue. The lung metastasis models were established by injecting hepa1-6 cells (1 × 10^6^) into the tail vein on the 14^th^ day to mimic the process of lung metastasis. On the 21 d, all mice were sacrificed and the lungs were extracted, followed by staining with Bouin's solution and H&E staining to visualize the lung metastatic state.

### FLI, MRI, and pharmacokinetics of OCT@ES *in vivo*

*In vivo* FLI was demonstrated using a NIRF imaging system (NightOWL II LB983, Germany) (Exc/Em = 748/780 nm). To assess the tumor-targeting performance of OCT@ES, DiR-labeled OCT@ES (30 mg/kg) were intravenously injected into the HCC mouse models. Then, the mice were anesthetized and imaged at numerous time points (0, 2, 4, 6, 8, 24, and 48 h). Afterward, the mice were sacrificed, followed by a dissection of the tumors and main organs for *ex vivo* FL imaging. For the MRI experiment *in vivo*, the MRI performance of the OCT@ES was demonstrated in HCC mouse models. A 3.0 T mouse MRI coil from Chenguang Medical Technology Company was used (CG-MUC40-H300-AS, Shanghai, China). The T_1_WI of the tumor area was imaged at different time points (0, 2, 4, 6, 8, and 24 h) after the intravenous injection of the OCT@ES (30 mg/kg). The T_1_WI parameters were set as follows: TR = 650 ms; TE = 11 ms; slice thickness = 1.5 mm; FOV = 80 mm. To assess the *in vivo* pharmacokinetics of the OCT@ES, the mice were injected intravenously with OCT@ES. Then, the mice were sacrificed at different time intervals (0, 15 min, 30 min, 1 h, 2 h, 4 h, 8 h, 12 h, 24 h, 48 h), followed by tumor and major organs (heart, liver, spleen, lung, kidney) extraction. All tissues were collected, weighed, dissolved with aqua regia (HCl: HNO_3_ = 3:1), evaporated, and re-dissolved in 1% HNO_3_. The Cu concentration was estimated by ICP-MS.

### Biosafety assay of OCT@ES *in vivo*

The bio-safety of the OCT@ES *in vivo* was evaluated on C57 BL6/J mice (male, 6-8 weeks). The mice were randomly assigned into six groups (control group and 1^st^, 3^th^, 7^th^, 14^th^, 28^th^ day group after injection of OCT@ES, n = 5 each group). On the 28^th^ day, all mice were sacrificed. Blood samples were collected for routine blood and biochemical examinations. The major organs (heart, liver, spleen, lung, and kidney) were collected for H&E analysis.

### Statistical analysis

All quantitative data are shown as the mean ± standard deviation (SD). Statistical analysis was performed using GraphPad 10.1 (La Jolla, CA, USA). A Student's unpaired or paired *t*-test was used to analyze the significant differences between the two groups. We used the one unpaired multiple *t*-test and analysis of variance (ANOVA) for the analysis of the differences between multiple groups. Statistical tests were double-sided, and values with *p* < 0.05 were considered statistically significant.

## Results and Discussion

### Characterization, cytotoxicity, and intracellular uptake of the OCT@ES

**Figure 1:**
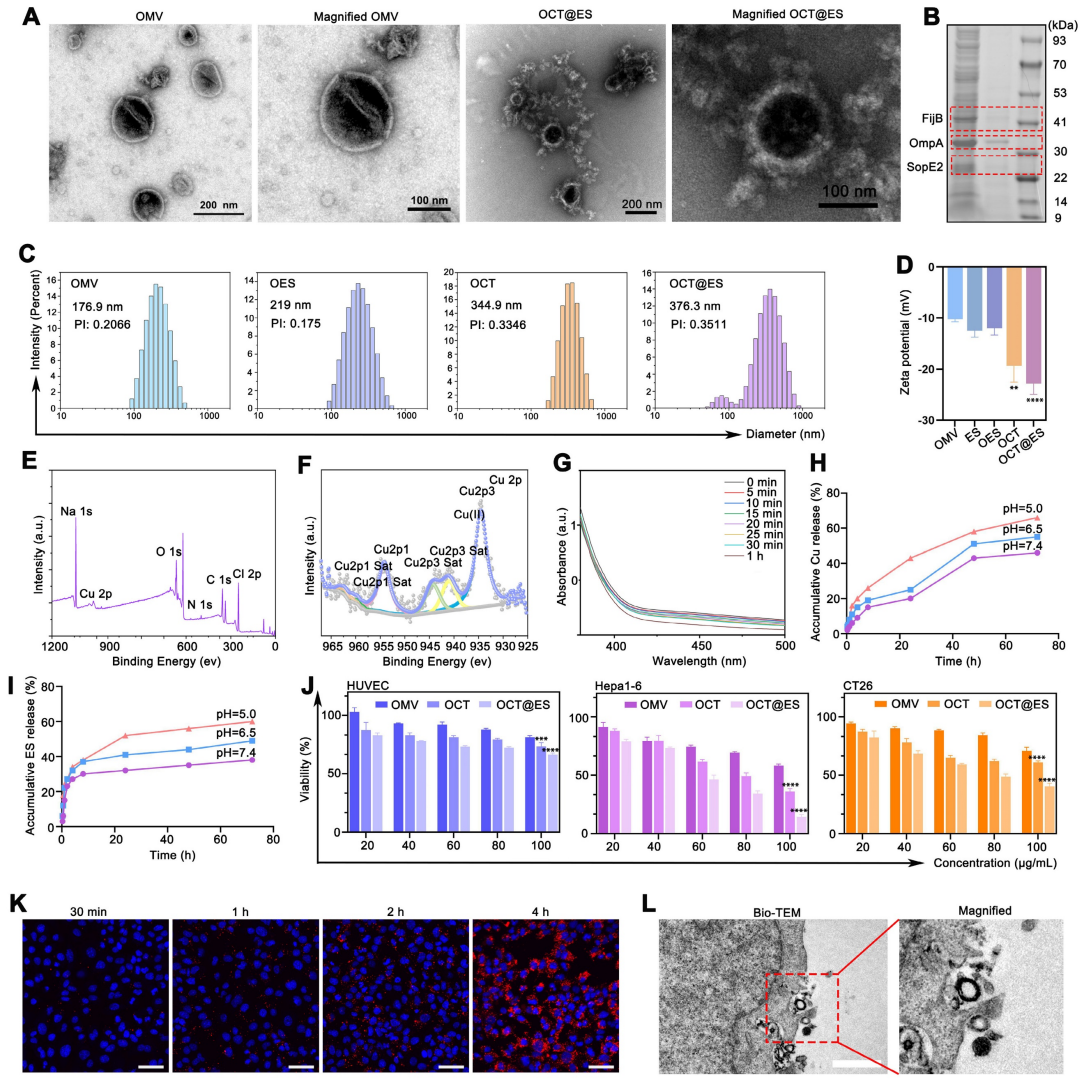
Characterization, cytotoxicity, intracellular uptake of OCT@ES. (A) TEM of OMV, OCT@ES, and elemental mapping of OCT@ES. Scale bar for OMV and OCT@ES: 200 nm, Scale bar for the magnified OMV and OCT@ES: 100 nm. (B) SDS-PAGE assay of OMV and OCT@ES. (C) DLS of OMV, OES, OCT, and OCT@ES. (D) Zeta potential of OMV, ES, OES, OCT, and OCT@ES. Data are shown as the mean values ± SD (n = 3). (E) XPS survey spectra and (F) XPS spectra of Cu2p. (G) The GPX-like activity of OCT@ES. (H) The accumulative Cu release in PBS with pH 5.0, pH 6.5, and pH 7.4. (I) The accumulative ES release in PBS with pH 5.0, pH 6.5, pH 7.4. (J) Cytotoxicity of OMV, OCT, and OCT@ES on HUVEC, hepa1-6 cells, and CT26 cells, respectively. Data are shown as the mean values ± SD (n = 5). (K) Intracellular uptake of DiI-labeled OCT@ES under CLSM. Scale bar: 50 μm. (L) The process of intracellular uptake of OCT@ES under Bio-TEM. Scale bar: 1 μm. All the statistical significance was analyzed by ANOVA, ∗∗∗∗ *p <* 0.0001, compared with the control group.

OMV was isolated from *E. coli* BL21 using a method described in a previous study 41. The encapsulation efficiency of ES was calculated to be 56% by HPLC, with a drug loading efficiency of 10%. According to the result of ICP-MS, the encapsulation efficiency of copper ions (Cu²⁺) varied based on the molar ratio of tannic acid (TA) to CuCl₂·2H₂O: 60% for 1:1, 71% for 1:2, and 72% for 1:3, respectively. The optimal molar ratio of TA to CuCl₂·2H₂O was determined to be 1:2, resulting in a Cu²⁺ loading efficiency of 3%. TA bound to the surface of OMVs via the phenolic hydroxyl groups, and the Cu²⁺ ions further stabilized the TA networks through coordination bonds 42. Transmission electron microscopy (TEM) images revealed that OMVs exhibited uniform, spherical, lipid-bilayered structures, while the OCT@ES nanoparticles displayed a core-shell structure, confirming the successful assembly of Cu-TA on the OMV surface **(Figure 1A)**. TEM mapping exhibited the distribution of various elements, including Cu, O, and S **(Figure S1)**. However, the TEM mapping images showed some background noise and strong Cu signals inside the OMVs, which may be due to the interference of contrast-enhancing reagents on the Cu distribution. As shown in **Figure 1B**, protein bands from OMVs were detected in the OCT@ES, confirming the incorporation of OMV components into the formulation. The average diameter of OMVs was 176.9 nm, as measured by dynamic light scattering (DLS)** (Figure 1C)**, which was consistent with previous reports for Gram-negative bacterial OMVs 43, 44. The hydrophobic ES was incorporated into the OMV lipid bilayer, and after surface modification with Cu-TA, the diameter increased to 344.9 nm for OCT and to 376.3 nm for OCT@ES. This confirmed the successful encapsulation of ES and the formation of a Cu-TA coating around the OMV surface **(Figure 1C)**. The result of zeta potential verified the same phenomenon **(Figure 1D)**. The OCT@ES displayed a more negative zeta potential than OMV due to the electronegativity of TA and ES. To evaluate the stability of OCT@ES, we measured its size over 14 days in both PBS and DMEM medium. The results showed minimal changes in particle size, indicating good stability **(Figure S2)**. The XRD pattern verified the amorphous structure of OMV and OCT@ES **(Figure S3)**. The elemental composition of the OCT@ES was detected further by XPS. As shown in the survey spectrum, Na, Cu, C, N, O, and Cl were presented in the spectrum of the OCT@ES **(Figure 1E)**. According to the Cu 2p XPS spectrum, Cu^2+^ remained the predominant ionic state in OCT@ES **(Figure 1F)**. According to previous reports, Cu^2+^ is paramagnetic which can serve as a positive contrast agent under T_1_-weighted MRI 45-47. Hence, we evaluated the hysteresis loop of OCT@ES by VSM. The results demonstrated the paramagnetic property of OCT@ES **(Figure S4)**. As revealed by FTIR **(Figure S5)**, the stretching vibrations of =C-H (3296 cm^-1^), and P-O (1057 cm^-1^) appeared in the absorption band of OMV, illustrating the presence of unsaturated aliphatic/aromatic hydrocarbons and poly-phospholipids on protein and phospholipid side chains 48. Although the FTIR pattern of OES did not show obvious change due to the low loading capacity of ES and the stealth caused by the strong OMV vibration band, the absorption intensity at 1314 cm^-1^ and 980 cm^-1^ was significantly increased due to the presence of C-H and C-N bond in ES, demonstrating the presence of ES on OMV. After coating of Cu-TA on the surface of OMV and OES, characteristic absorption bands of TA (C=0, C=C, C-O) at 1717 cm^-1^, 1444 cm^-1^, 1201 cm^-1^ appeared in the absorption band of both OCT and OCT@ES, showing the successful coating of Cu-TA on OMV surface. Notably, the absorption of OCT@ES exhibited a redshift phenomenon at 1322 cm^-1^ and 1036 cm^-1^, which might be ascribed to the coordination between Cu^2+^ and the oxygen-containing group from TA. The intracellular free Cu ions can also be scavenged by GSH, a prominently expressed endogenous thiol-containing copper chelator, thus compromising the intensity of cuproptosis 26. To assess the ability of OCT@ES to consume intracellular GSH, DTNB was chosen as an indicator to measure GSH consumption by measuring the characteristic UV-Vis absorption peak at 412 nm 49. As revealed by** Figure 1G**, a time-dependent decrease in GSH content was identified, confirming that the OCT@ES was capable of consuming GSH and sensitizing cells to cuproptosis. Cu-TA was also reported to possess pH responsiveness since the coordination bond in Cu-TA tends to degrade in an acidic environment 50. To confirm the pH responsiveness of OCT@ES, we evaluated the release behavior of Cu and ES in PBS solutions at different pH values (5.0, 6.5, and 7.4). The results showed distinct acid-responsive release behavior of Cu and ES, indicating that Cu and ES could be released in tumor cells accurately because the intracellular lysosome pH of tumor cell is 5.0 (**Figure 1H and 1I**) 51, 52. Additionally, Cu-TA and ES slowly detached from OCT@ES in the slightly acidic tumor microenvironment (TME), allowing for the exposure of OMV and recruitment of T-cells for a tumoricidal effect.

The bio-safety and tumor-killing effects of OMV, OCT, and OCT@ES were further evaluated at the cellular level. As revealed in **Figure 1J**, Cell counting kit-8 (CCK-8) results showed that OMV, OCT, and OCT@ES all reduced the viability of HUVEC in a concentration-dependent manner, but the viability remained above 70% in all treatment groups, indicating the biocompatibility of OCT@ES in non-cancerous cells. **Figure 1J and Figure S6** manifested that OMV, OCT, and OCT@ES significantly reduced the viability of multiple tumor cell lines (Hepa1-6, CT26, B16, and 4T1) in a dose-dependent manner. Specifically, OCT displayed stronger cytotoxicity than OMV, suggesting that the Cu-TA coating enhanced the tumoricidal effect. Notably, OCT@ES exhibited the strongest cytotoxicity, with IC50 values of 76.27 μg/mL for CT26 cells, 55.28 μg/mL for Hepa1-6 cells, 118.8 μg/mL for 4T1 cells, and 83.54 μg/mL for B16 cells. These results indicated that CT26 and Hepa1-6 cells were more sensitive to OCT@ES nanoparticles. Therefore, we chose CT26, and Hepa1-6 tumor cells to evaluate the following *in vitro* and/or* in vivo* antitumor effect and explore corresponding therapeutic mechanisms.

Effective intracellular uptake of OCT@ES by tumor cells is a prerequisite for achieving desirable antitumor efficacy. To assess the intracellular uptake of OCT@ES by tumor cells, we used DiI-labeled OCT@ES and evaluated uptake by CLSM.** Figure 1K** displayed the red FL intensity increased over time in Hepa1-6 cells, indicating efficient internalization of DiI-labeled OCT@ES. This phenomenon was also verified by FCM as demonstrated in **Figure S7**. Furthermore, we determined the cellular uptake manner of hepa1-6 cells for OCT@ES via Bio-TEM **(Figure 1L)**. We observed that hepa1-6 cells engulfed OCT@ES by endocytosis.

To summarize, the OCT@ES was successfully synthesized and characterized. The nanoparticles exhibited spherical, lipid-bilayered structures, favorable nanoscale size, GSH consumption activity, pH-responsive release, biocompatibility, and efficient tumoricidal effects. These properties make OCT@ES a promising candidate for targeted cancer therapy.

### The mechanistic study of CPApoptosis triggered by OCT@ES *in vitro*

**Figure 2:**
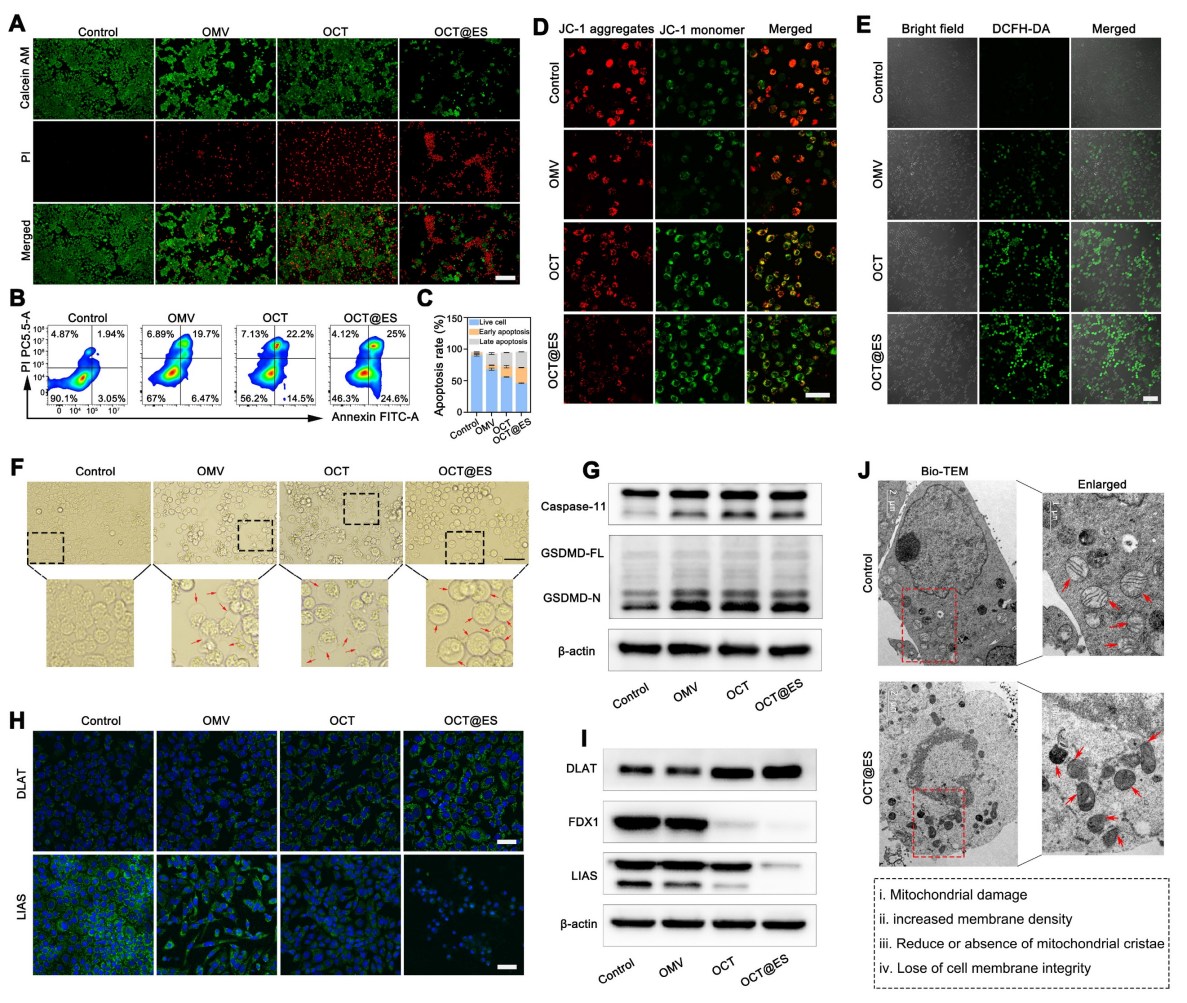
The mechanistic study of CPApoptosis. (A) Live/dead cells staining of hepa1-6 cells after treatments with PBS, OMV, OCT, and OCT@ES, respectively under a FL microscope. Scale bar: 100 μm. (B, C) Apoptotic assay of hepa1-6 cells under FCM after various treatments and corresponding quantitative analysis. Data are shown as the mean values ± SD (n = 3). (D) CLSM of mitochondrial potential change in different treatment groups. Scale bar: 25 μm. (E) CLSM of DCFH-DA expression in different treatment groups. Scale bar: 100 μm. (F) Bright field of cell morphology in different treatment groups under a microscope. Scale bar: 100 μm. (G) WB analysis of caspase 11 and GSDMD expression in the non-canonical pyroptosis signaling pathway in different treatment groups. (H) CLSM of DLAT and LIAS expression in different treatment groups. Scale bar: 50 μm. (I) WB analysis of DLAT, FDX1, and LIAS expression in different treatment groups. (J) Bio-TEM images of hepa1-6 cells in the control and OCT@ES groups. Scale bar for the left Bio-TEM: 2 μm, Scale bar for the right enlarged bio-TEM: 1μm.

Upon introduction into tumor cells, the coordination bond between Cu²⁺ and tannic acid (TA) in OCT@ES was broken, leading to the gradual detachment of Cu-TA from the OMV surface. Cu^2+^ was reduced to Cu^+^ in the presence of TA for ROS generation. ROS was believed to attack mitochondria, causing mitochondrial dysfunction and activating the oxidative apoptotic pathway 39. To investigate the antitumor effects of OCT@ES, we first performed live/dead cell staining. The results showed that treatment with both OMV and OCT moderately increased the number of PI-positive dead cells compared to the control, with a majority of Calcein AM-positive cells still present. In contrast, the OCT@ES treatment notably increased the number of PI-positive cells and significantly reduced the proportion of live cells, confirming that OCT@ES exhibited the strongest tumor-killing effect**(Figure 2A)**. It was also found that the apoptotic ratio of hepa1-6 cells in all treated groups was significantly higher than that in the control group, among which the OCT@ES treated group exhibited most apoptotic cells with an apoptotic ratio over 50% **(Figure 2B and 2C)**. The results inferred that OCT@ES effectively activated the apoptotic pathway in hepa1-6 cells. To explore the biological mechanisms underlying OCT@ES-induced apoptosis, we focused on oxidative stress and mitochondrial dysfunction, both of which are linked to copper ions. First, we used the JC-1 fluorescent probe to assess mitochondrial membrane potential (MMP). The result of FCM showed that both OCT and OCT@ES treatment significantly shifted the JC-1 aggregates to JC-1 monomers, indicating a loss of MMP and mitochondrial dysfunction** (Figure S8A and S8B)**. The OCT@ES-induced MMP loss was also demonstrated by CLSM, which showed a clear shift from red to green fluorescence in the OCT@ES-treated cells, signifying mitochondrial dysfunction** (Figure 2D)**. Afterward, we detected the cellular oxidative stress level using a DCFH-DA probe. As shown in **Figure 2E**, CLSM images showed that both the OCT and OCT@ES groups had a large number of DCFH-DA positive tumor cells, indicating increased ROS generation. This effect was likely due to TA/GSH-mediated Cu⁺ generation followed by a Fenton-like reaction, which produces excessive ROS in the presence of intracellular H₂O₂. The phenomenon was also validated by FCM analysis **(Figure S8C and S8D)**. In a word, OCT@ES could effectively induce tumor cell apoptosis mainly through TA/GSH-mediated Cu^+^ generation and the subsequent Cu^+^-induced Fenton-like reaction, whereas OMVs alone had little impact on ROS generation in tumor cells.

In addition to inducing apoptosis, the OCT@ES treatment was also found to induce pyroptosis as observed under bright-field microscopy. Pyroptotic cell morphology, characterized by swelling and the formation of large bubbles, was evident following treatment with OMV, OCT, and OCT@ES as shown in **Figure 2F**. In contrast, the control group maintained intact cell morphology. This finding aligns with previous studies showing that OMVs act as vehicles to deliver lipopolysaccharides (LPS) into the cytosol 41, 53, 54. The cytosolic LPS activates human caspase-4/5 or mouse caspase-11, initiating the non-canonical pathway of pyroptosis by cleaving GSDMD into GSDMD-N 55, 56, which forms pores in the plasma membrane and induces cell swelling and cytolysis. The release of cytosolic substances and inflammatory molecules, including TAAs, CRT, HMGB1, IL-1β, and LDH, stimulates dendritic cell (DC) maturation 57. The OCT and OCT@ES group exhibited more pyroptotic cells as compared to the OMV group, which could be attributed to the elevated Cu²⁺ levels inside the tumor cells, increasing intracellular osmolarity and enhancing the severity of pyroptosis. Encouraged by the above phenomenon, the mechanism of OCT@ES-induced pyroptosis was explored by detecting the expression of pyroptosis-related proteins, including caspase-11, GSDMD-FL, and GSDMD-N. As expected, the OMV, OCT, and OCT@ES-treatment increased the expression level of caspase-11 and GSDMD-N **(Figure 2G and Figure S9A)**. These results confirm that OCT@ES induces tumor cell pyroptosis through the LPS-triggered non-canonical pathway.

Recent studies have highlighted that Cu ions are responsible for a novel form of cell death known as cuproptosis, but the Cu ions must be precisely transported into the mitochondria to initiate this process 21. In our study, ES in OCT@ES played a key role in triggering cuproptosis, as ES and Cu²⁺ dissociated from OCT@ES formed a Cu-ES complex via coordination bonds. This complex then actively targeted the mitochondria. Once inside, Cu²⁺ dissociated from the Cu-ES complex and bound to FDX1, a mitochondrial reductase involved in Fe-S cluster formation, which reduced Cu²⁺ to Cu⁺. The Cu⁺ then caused DLAT aggregation, leading to proteotoxic stress and cuproptosis. TA dissociated from OCT@ES also contributed to cuproptosis by depleting intracellular GSH, a copper chelator that can limit the intensity of cuproptosis. The intracellular GSH-consuming ability of OCT@ES was evidenced in **Figure S10**. Based on the aforementioned hypothesis, we first conduced IF staining to observe the expression of two cuproptosis-related proteins, DLAT and LIAS **(Figure 2H)**. CLSM images showed that DLAT was minimally expressed and evenly distributed around the nucleus in the control and OMV-treated groups. In the OCT treatment group, moderate DLAT aggregation was observed, with scattered DLAT foci, while the OCT@ES group exhibited pronounced DLAT aggregation and increased DLAT foci. Conversely, the mitochondrial iron-sulfur cluster protein LIAS was disrupted in both the OCT and OCT@ES groups. The results of IF staining confirmed that Cu-TA anchored on the OMV surface was responsible for the induction of cuproptosis, while the addition of Cu ion transporter, ES, amplified the intensity of cuproptosis by precisely transporting Cu ion into the mitochondria. WB analysis confirmed these findings, showing that the control and OMV-treated groups had negligible effects on DLAT aggregation and the loss of iron-sulfur cluster proteins (LIAS and FDX1), whereas OCT and OCT@ES treatment significantly induced cuproptosis, as evidenced by increased DLAT aggregation and reduced LIAS and FDX1 levels **(Figure 2I and Figure S9B)**. Finally, bio-transmission electron microscopy (Bio-TEM) analysis further confirmed these results. As exhibited in **Figure 2J**, Hepa1-6 cells treated with OCT@ES displayed significant cell membrane rupture and organelle damage consistent with pyroptosis, as well as mitochondrial shrinkage, chromatin condensation, and the loss of mitochondrial cristae, characteristic features of cuproptosis.

To conclude, the as-synthesized OCT@ES was a ternary CPApoprosis nano-actuator that could trigger cell apoptosis by oxidative stress, pyroptosis by non-canonical caspase-11 pathway, and cuproptosis by proteotoxic stress (**Figure S9C**). This multifunctional approach demonstrates the potential of OCT@ES in effective cancer therapy by triggering robust immune responses and tumor cell destruction.

### The evaluation of ICD induction and DC maturation *in vitro*

**Figure 3:**
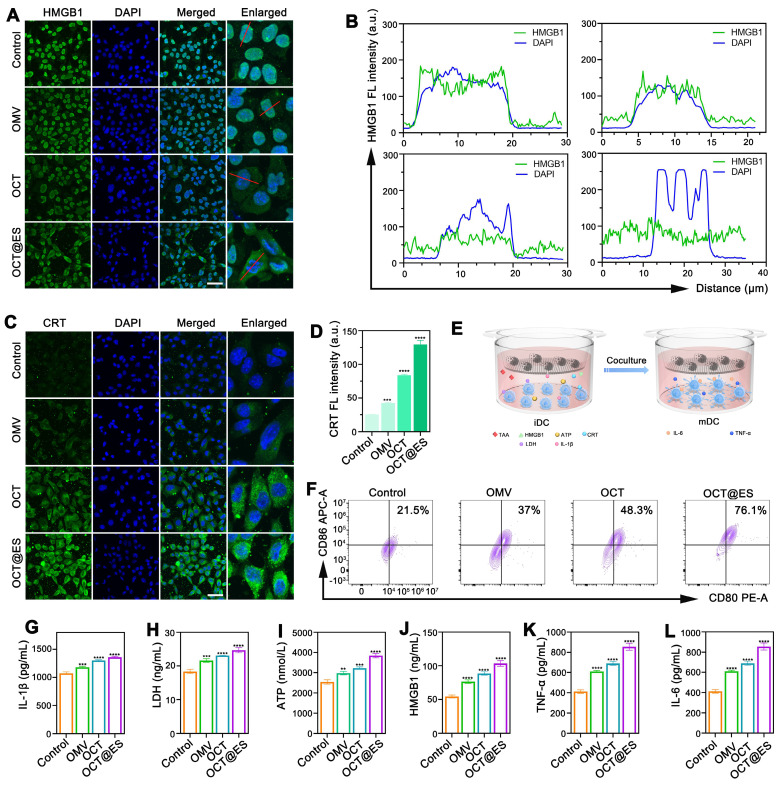
The evaluation of ICD induction and DC maturation. (A) CLSM of the HMGB1 expression and (B) the FL co-localization of DAPI and HMGB1. Scale bar: 50 μm. (C) CLSM of the CRT expression and (D) the FL quantitative analysis of CRT. Data are shown as the mean values ± SD (n = 3). Scale bar: 50 μm. (E) The schematic illustration of hepa1-6 cell and JAWSII cells on a coculture system. (F) The FCM analysis of DC maturation. (G, H, I, J, K, L) The ELISA assay of IL-1β, LDH, ATP, HMGB1, TNF-α, and IL-6. Data are shown as the mean values ± SD (n = 3). All the statistical significance was analyzed by ANOVA, ∗∗ *p <* 0.01, ∗∗∗ *p <* 0.001, ∗∗∗∗ *p <* 0.0001, compared with the control group.

Many studies demonstrated that apoptosis, pyroptosis, and cuproptosis are all immunogenic, which could cause immunogenicity and adjuvanticity for enhancing the efficacy of cancer immunotherapy by exposing “eat me” signals on dying tumor cell surface or releasing soluble immunogenic factors, ultimately bolstering DC maturation 12. Specifically, the disruption of cell membrane integrity in pyroptosis facilitates the release of substantial quantities of immunogenic cell death (ICD) factors, leading to effective DC maturation. Hence, in this part, we evaluated the intensity of ICD by measuring the expression of HMGB1 and CRT **(Figure 3A, 3B, 3C, and 3D)**, which are commonly used to assess immunogenicity 58. The results of HMGB1 IF staining showed that HMGB1 mainly overlapped with the DAPI-stained nucleus with little migration into the cytosol in the control group, while in the OMV-treated group, there was a noticeable reduction in nuclear HMGB1 density, accompanied by an increase in its distribution in the cytoplasm. After OCT treatment, the translocation of HMGB1 from the nucleus to the cytosol became more distinct. In the OCT@ES-treated group, HMGB1 was predominantly found in the cytosol, with minimal presence in the nucleus, indicating effective HMGB1 migration—a hallmark of ICD. For CRT, the intracellular fluorescence intensity was significantly higher in the OMV, OCT, and OCT@ES treatment groups compared to the control. Notably, the OCT@ES group exhibited the brightest CRT fluorescence, signifying that CRT was exposed on the surface of dying tumor cells, acting as an “eat me” signal to facilitate DC maturation. This result further supports that OCT@ES induced the most robust ICD.

To assess whether OCT@ES could trigger DC maturation, we established a transwell system using immature JAWSII DCs and Hepa1-6 cells. We then analyzed DC maturation via FCM and tested various soluble immunogenic and inflammatory factors in the culture medium, including ATP, HMGB1, IL-1β, LDH, TNF-α, and IL-6, using ELISA **(Figure 3E)**. First, we evaluated the effects of OMV, OCT, and OCT@ES on the maturation of JAWSII cells via FCM. The results showed that all treatments slightly increased the proportion of matured DCs compared to the control** (Figure S11)**. However, when JAWSII cells were cultured on the transwell system with tumor cells, all treatment groups induced a significant increase in the proportion of matured DCs (CD11c^+^CD80^+^CD86^+^) (**Figure 3F and Figure S12**). Notably, the OCT@ES-treated group showed the highest increase, with a 3.5-fold greater proportion of matured DCs compared to the control. This was consistent with the enhanced ICD induced by OCT@ES, as reflected in HMGB1 translocation and CRT exposure. Next, we measured the levels of pyroptosis-related factors—IL-1β and LDH—via ELISA. Both IL-1β and LDH were significantly elevated in the three treatment groups compared to the control, confirming the initiation of pyroptosis induced by OMV **(Figure 3G, 3H)**. Additionally, the levels of ATP and HMGB1, which are critical for DC maturation, were increased in the culture medium, further supporting the occurrence of ICD **(Figure 3I, and 3J)**. Finally, we assessed the secretion of TNF-α and IL-6, which are indicative of matured DCs. These two factors were significantly elevated in all treatment groups, with the OCT@ES group showing the highest levels of TNF-α and IL-6, indicating that OCT@ES effectively induced the transformation of immature DCs into functional, matured DCs** (Figure 3K, and 3L)**.

### FLI, pharmacokinetics, and MRI of OCT@ES *in vivo*

In cellular experiments, the OCT@ES was proven to be engulfed by hepa1-6 cells via endocytosis in a time-dependent manner, allowing for the initiation of CPApoptosis intracellularly. However, the complexity of *in vivo* systems is much greater than that of cell cultures. To track the* in vivo* distribution of OCT@ES, we labeled it with DiR, a commonly used dye for *in vivo* fluorescence imaging, and traced its distribution in Hepa1-6 tumor-bearing mice. As shown in **Figure S13A and 13B**, the FL signal at the tumor site gradually increased in a time-dependent manner, and then peaked at 24^th^ h, showing the strongest FL signal intensity. The signal intensity slightly decreased at 48^th^ h, suggesting that DiR-labeled OCT@ES effectively accumulated at the tumor site and remained there for 48 hours, potentially due to the EPR effect facilitated by its nanoscale size. Of note, DiR-labeled OCT@ES largely accumulated at the liver organ. After the 48^th^, all mice were sacrificed to obtain tumor tissue and major organs (heart, liver, spleen, lung, kidney) for *ex vivo* FL imaging. As shown in **Figure S13C and 13D**, the liver exhibited the strongest FL signal intensity, followed by the spleen, lung, tumor, kidney, and heart. The reason why the liver and the spleen displayed such intense FL signals might be due to the abundant blood supply of the liver and spleen, and the MPS residing in the liver environment 59. However, OCT@ES demonstrated suboptimal tumor targeting, which may be attributed to the passive tumor-targeting mechanism based solely on the EPR effect. In future studies, we plan to enhance the tumor-targeting ability of OMVs by functionalizing them with tumor-specific ligands, such as peptides or antibodies, for active targeting.

To further detect the *in vivo* accumulation and distribution of Cu elements in tumor tissues and major organs, ICP-MS was conducted to measure the Cu content of each tissue at different time intervals. The results showed that Cu preferentially accumulates in the lung, heart, and liver, which might be due to the rapid blood supply and then captured by the reticuloendothelial system, followed by metabolization and elimination by the kidney over time** (Figure S13E)**. Notably, the maximum content of Cu was found in the tumor at the 8^th^ hour. This discrepancy between the ICP and FL results can be attributed to the differing pharmacokinetics of copper ions and DiR dye, which metabolize and distribute in the body in different ways.

Based on the fact that Cu^2+^ is paramagnetic which can serve as a positive contrast agent, we evaluated the MR imaging performance of the OCT@ES due to the presence of Cu^2+^. As expected, the gray scale value of the tumor increased over time, as visualized in **Figure S13F**. The tumor site exhibited the brightest signal at 8^th^ h after intravenous injection of OCT@ES, which was in accordance with the result of Cu pharmacokinetic *in vivo*. Then, the MRI gray value decreased at 24^th^ h. The reason for this phenomenon might be that Cu^2+^ was gradually transformed into Cu^+^ in the presence of TA and GSH, diminishing the amount of Cu^2+^ available for T_1_-weight MRI. These findings suggest that OCT@ES provides a promising MR imaging tool for in vivo tracking of copper kinetics at the tumor site, offering a theranostic platform for cancer management.

### The antitumor effect induced by OCT@ES in combination with αPD-L1 on HCC and colorectal cancer models

**Figure 4:**
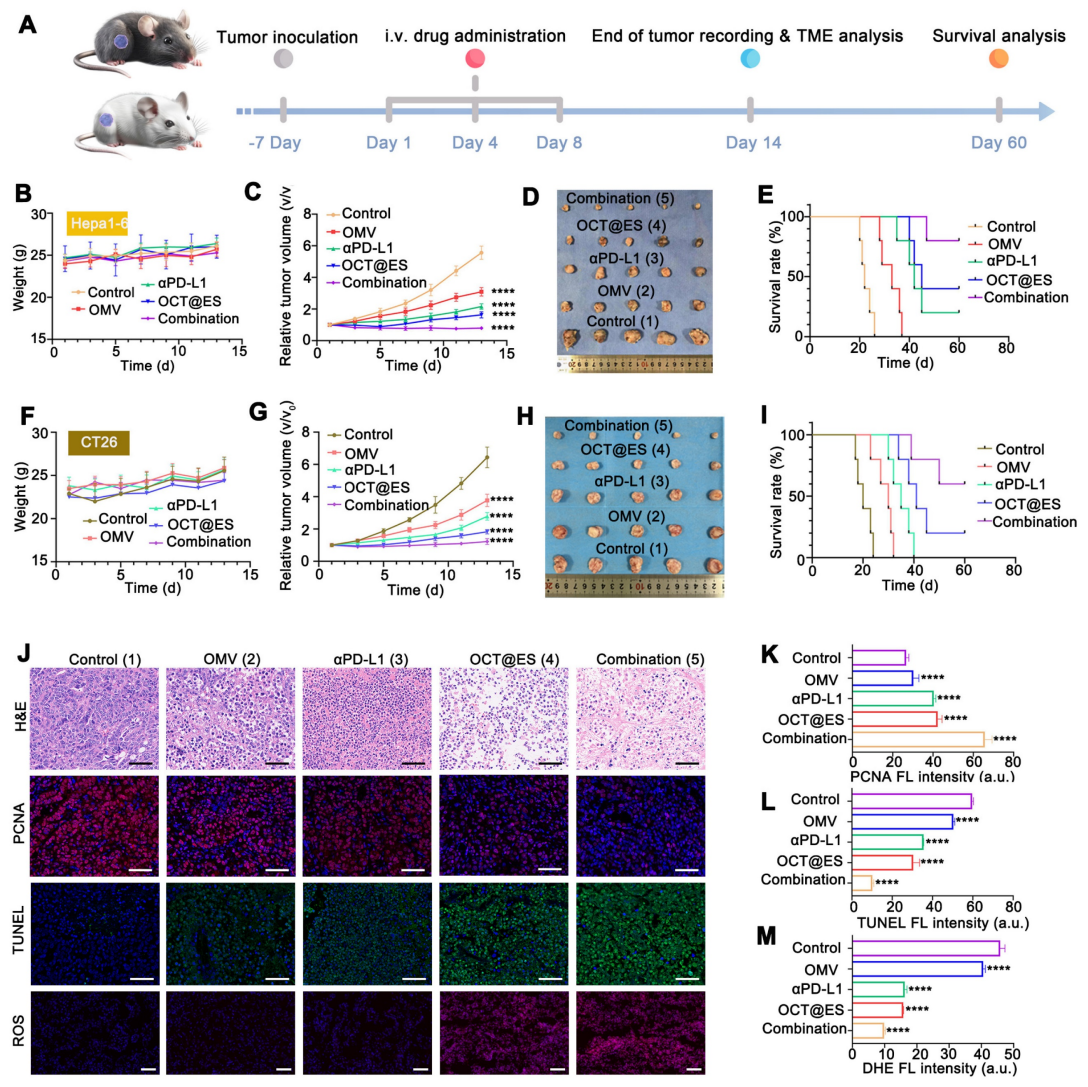
Evaluation of antitumor effect *in vivo*. (A) The schematic illustration of the therapeutic paradigm on hepa1-6 mouse model and CT26 mouse model. (B) Average body weight change of hepa1-6 mouse model during the observation duration. Data are shown as the mean values ± SD (n = 5). (C) The relative tumor growth curve of the hepa1-6 mouse model in different treatment groups. Data are shown as the mean values ± SD (n = 5). (D) The digital photograph of tumors extracted from the hepa1-6 mouse models at sacrifice on the 14^st^ day. (E) The long-term survival observation in the hepa1-6 mouse models with different treatment. Data are shown as the mean values ± SD (n = 5). (F) Average body weight change of CT26 mouse model during the observation duration. Data are shown as the mean values ± SD (n = 5). (G) The relative tumor growth curve of the CT26 mouse model in different treatment groups. Data are shown as the mean values ± SD (n = 5). (H) The digital photograph of tumors extracted from the CT26 mouse models at sacrifice on the 14^st^ day. (I) The long-term survival observation in the CT26 mouse models with different treatment. Data are shown as the mean values ± SD (n = 5). (J) The representative H&E, PCNA, TUNEL, and DHE staining of tumor slices after different treatments. Scale bar: 50 μm. (K, L, M) The quantitative FL analysis of PCNA, TUNEL, and DHE. Data are shown as the mean values ± SD (n = 3). All the statistical significance was analyzed by ANOVA, ∗∗∗∗ *p <* 0.0001, compared with the control group.

In cellular experiments, OCT@ES demonstrated effective tumoricidal effects on both Hepa1-6 and CT26 cells. However, it has been reported that cuproptosis can induce PD-L1 expression on tumor cells, potentially promoting immune evasion 60. Therefore, combining OCT@ES with the immune checkpoint inhibitor, anti-PD-L1 antibody (αPD-L1), may enhance therapeutic efficacy. To evaluate the *in vivo* therapeutic potential of this combinational therapy, we established two immunologically cold tumor mouse models, one using Hepa1-6 cells and the other using CT26 cells. The treatment protocol is depicted in **Figure 4A**. All mice were divided into five groups: the control, OMV, αPD-L1, OCT@ES, and the combination group (αPD-L1 + OCT@ES). As shown by the results from the Hepa1-6 mouse model, the average body weight of the mice remained stable, suggesting no significant* in vivo* toxicity **(Figure 4B)**. In comparison to the control group, which exhibited rapid tumor growth, the OMV and αPD-L1 treatments moderately suppressed tumor growth. However, the tumor volume was significantly reduced in the OCT@ES-treated group, likely due to the synergistic tumor-killing effects of cuproptosis, pyroptosis, and apoptosis **(Figure 4C and Figure S14A)**. Importantly, the combination of αPD-L1 with OCT@ES further enhanced therapeutic efficacy, as evidenced by the slowest tumor growth rate and prolonged survival **(Figure 4C, and 4E)**. After the 14^th^ day, all mice were sacrificed, followed by tumor extraction to obtain digital photos and tumor weighing. As shown in **Figure 4D and Figure S14B**, tumors in the combination group were the smallest which was consistent with the outcomes of the tumor volume growth curve. Afterward, we collected tumor tissue from hepa1-6 mouse models after sacrifice and sent them for H&E staining and IF staining of PCNA, TUNEL, and ROS (**Figure 4J, 4K, 4L, and 4M**). The results of H&E staining, PCNA staining, and TUNEL fluorescence staining results further verified that the combinational treatment could induce significant histological damage, serious cell proliferation inhibitory activity, and extensive apoptosis of hepa1-6 tumors, confirming that OCT@ES could activate tumor cell apoptosis signaling pathway *in vivo* which could be further augmented by αPD-L1. DHE staining revealed increased ROS generation in the OCT@ES and combination treatment groups, consistent with the in vitro results, indicating that the Cu-TA component of OCT@ES contributed to ROS production, which initiated apoptosis. Furthermore, we collected the major organs from mice after different treatments and did H&E staining. The results showed that all major organs displayed no abnormalities in terms of structures and histomorphology, indicating the biosafety of all treatment strategies** (Figure S15)**.

To evaluate whether the antitumor effects of the combinational therapy could be generalized, we established a CT26 mouse model, which is also immunologically silent. The tumor volume growth and relative tumor volume change curves showed similar therapeutic outcomes to the Hepa1-6 model **(Figure S14C and 4G)**. In all treatment groups, tumor growth was inhibited compared to the control group. The OMV and αPD-L1 groups showed moderate tumor growth suppression, while OCT@ES treatment significantly inhibited tumor growth. The addition of αPD-L1 further amplified this effect. During the observation, the body weight of the mice in each group fluctuated in the normal range after drug administration, which indicates the excellent biosafety of all treatments **(Figure 4F)**. The images and weights of the extracted tumor tissues from the combinational therapy-treated group seemed to be the smallest, demonstrating the synergistical therapeutic effect induced by αPD-L1 and OCT@ES **(Figure 4H and S14D)**. The survival rate of the CT26 mouse models treated by the combinational therapy was also significantly prolonged **(Figure 4I)**, demonstrating the efficient tumor suppression effect of the combinational therapy on CT26 mouse models.

### The mechanistic study of CPApoptosis *in vivo*

**Figure 5:**
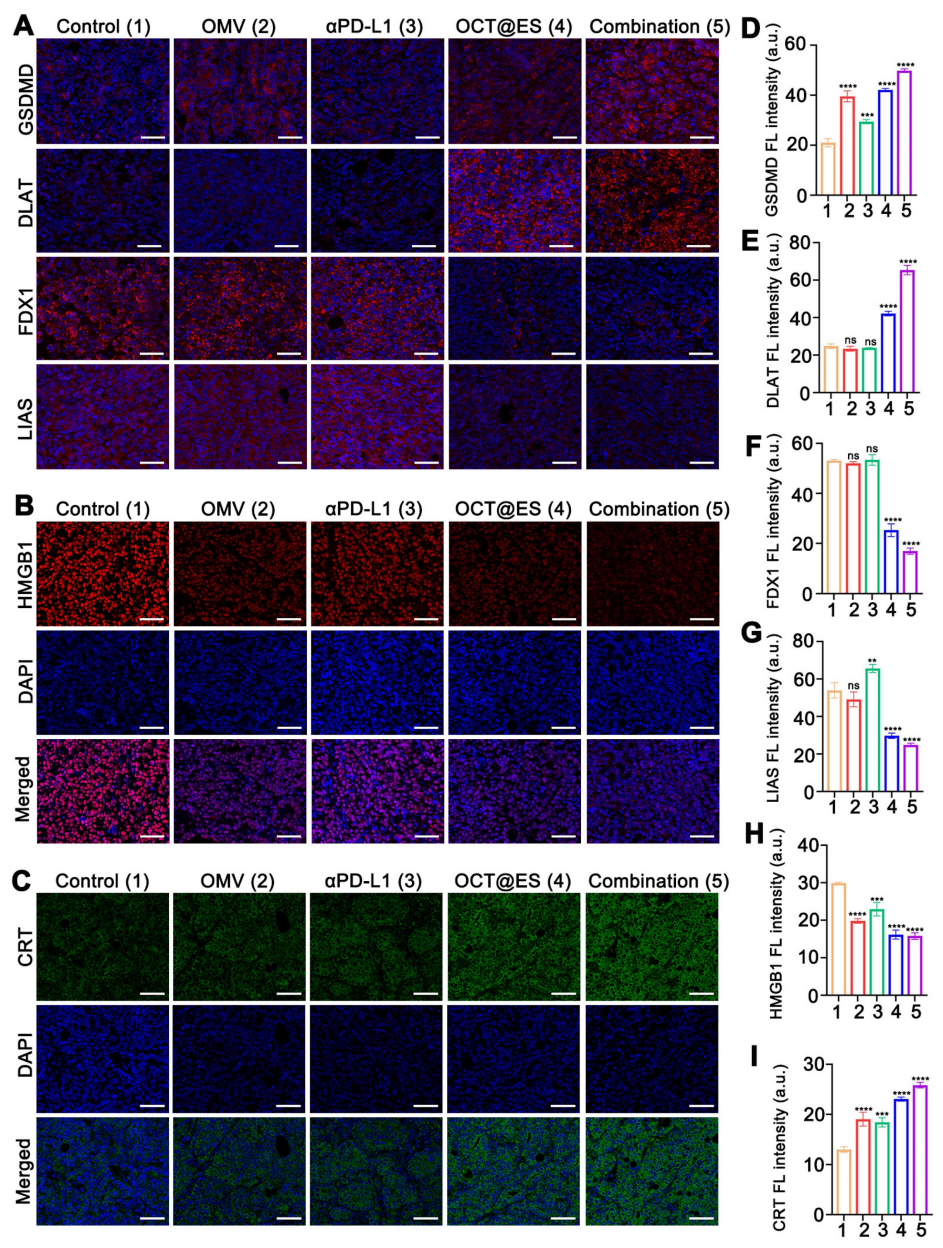
The mechanistic study of CPApoptosis *in vivo*. (A) IF staining of GSDMD, DLAT, FDX1, and LIAS in tumor sections after various treatments. Scale bar: 50 μm. (B) IF staining of HMGB1 and (C) CRT in tumor sections after various treatments. Scale bar: 50 μm. (D, E, F, G, H, I) Quantitative FL analysis of GSDMD, DLAT, FDX1, LIAS, HMGB1, and CRT. Data are shown as the mean values ± SD (n = 3). All the statistical significance was analyzed by ANOVA, ∗∗ *p <* 0.01, ∗∗∗ *p <* 0.001, ∗∗∗∗ *p <* 0.0001, ns, not significant, compared with the control group.

To elucidate whether this antitumor effect resulted from pyroptosis and cuproptosis, we continued to examine histological expression of GSDMD, DLAT, LIAS, FDX1 in HCC tumor tissues via IF assay **(Figure 5A, 5D, 5E, 5F, and 5G)**. Regarding pyroptosis-related biomarkers, the IF staining showed that the tumor tissues isolated from the mice treated with OMV or OCT@ES exhibited considerable GSDMD expression compared to the control group and the αPD-L1 treated group with negligible GSDMD positive signals, which was consistent with the *in vitro* study, indicating that the OMV was the key component to trigger pyroptosis.

The combinational treatment significantly enhanced the expression of GSDMD, which might be ascribed to the intensive immune activation that in turn amplified the intensity of pyroptosis. For cuproptosis-related biomarkers, IF staining of DLAT showed that both the OCT@ES and combinational therapy groups had markedly higher FL intensity for DLAT compared to the control, OMV, and αPD-L1-treated groups. This suggests that more lipoylated protein aggregation occurred due to the presence of Cu-TA and ES in OCT@ES. Furthermore, downregulation of FDX1 and LIAS proteins was observed in the tumor tissues treated with OCT@ES and combinational therapy, further confirming that Cu-TA and ES are essential for initiating cuproptosis *in vivo*, consistent with our in vitro results. Taken together, these findings indicate that OCT@ES upregulates key markers involved in multiple cell death pathways, including pyroptosis and cuproptosis, *in vivo*.

ROS-induced apoptosis, OMV-induced pyroptosis, and Cu-induced cuproptosis were previously reported to be immunogenic, leading to immune stimulation and DC maturation by releasing abundant DAMPs 5. To assess whether CPApoptosis induced by OCT@ES triggered an immune response in vivo, we examined the expression of DAMPs (CRT and HMGB1) in tumor tissues via IF assays **(Figure 5B, 5C, 5H, and 5I)**. Similar to the result of *in vitro* study, the OMV and αPD-L1 treated group showed moderate expression level of CRT and little HMGB1 translocation from the nucleus to the cytosol. In contrast, the OCT@ES and combination therapy groups exhibited distinct CRT exposure on the cell surface and significant HMGB1 migration to the cytosol. These findings confirm that OCT@ES-induced CPApoptosis is highly immunogenic, leading to the release of DAMPs that can stimulate immune activation. This process is conducive to enhancing the effectiveness of immune checkpoint inhibitors, such as αPD-L1, through synergistic immunotherapy.

### The evaluation of tumor immune microenvironment

**Figure 6:**
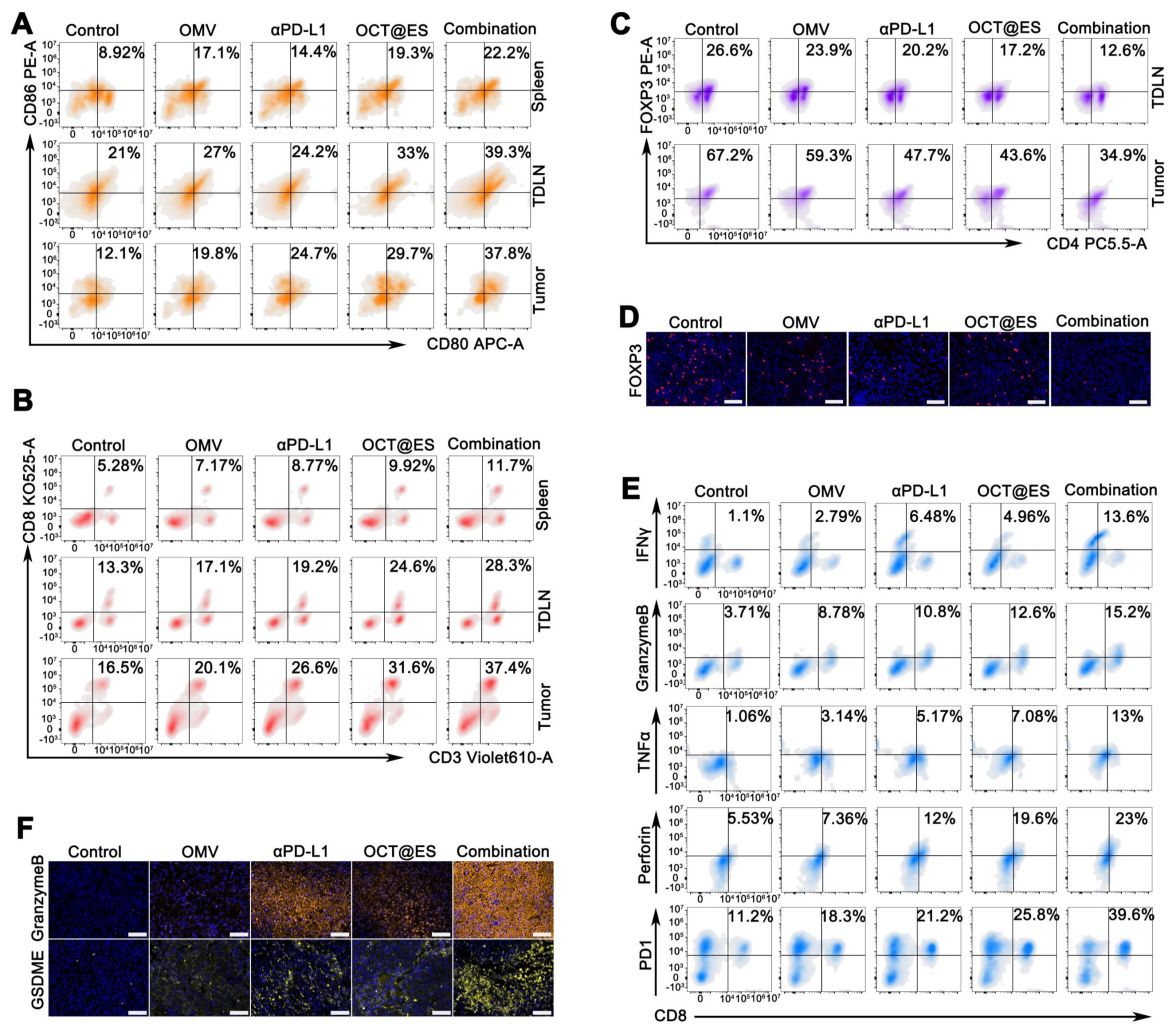
The evaluation of tumor immune microenvironment. (A) Representative FCM results of matured DCs (CD11c^+^CD80^+^CD86^+^) within spleens, TDLNs, and tumors after different treatments. (B) Representative FCM results of CTL (CD3^+^CD8^+^) within spleens, TDLNs, and tumors after different treatments. (C) Representative FCM results of Treg (CD3^+^CD4^+^FOXP3^+^) within the TDLNs, and tumors after different treatments. (D) IF staining of FOXP3 in tumor sections after different treatments. Scale bar: 50 μm. (E) Representative FCM results of T-cell subtypes (CD8^+^IFNγ^+^, CD8^+^granzymeB^+^, CD8^+^TNFα^+^, CD8^+^perforin^+^, CD8^+^PD1^+^) within the tumors after different treatments. (F) IF staining of granzyme B and GSDME in tumor sections after different treatments. Scale bar: 50 μm.

Since the CPApoptosis was confimed to be immunogenic, the population of crucial immune cells (DC and T) in the spleen, TDLNs, and the primary tumor tissues that contribute to antitumor immunity were assessed by FCM. The gating plots (FSC-A/SSC-A and FSC-A/FSC-H) for lymphocyte analysis in the tumor sites, TDLNs, and the spleens were provided in **Figure S16**. As proved by *in vitro* study, tumor cells that underwent CPApoptosis can release abundant DAMPs which enhance tumor immunogenicity and promote the maturation of naive DCs. In the *in vivo* study, the OMV and αPD-L1 treatment moderately primed the population of matured DCs in all tissues as compared with the control **(Figure 6A and Figure S20A)**. The number of matured DCs obtained from the spleen, TDLNs, and tumor tissue receiving OCT@ES exhibited 2.16-fold, 1.57-fold, and 2.45-fold higher than that in the control group. The DC maturation rate in each tissue was further augmented by 2.49-fold, 1.87-fold, and 3.12-fold respectively after receiving the combinational treatment, which might be due to the synergistic immune activation induced by OCT@ES and αPD-L1. This robust DC maturation is seen as the key to mediating the downstream adaptive immunity by cross-priming effector T cells and facilitating T cell proliferation to initiate anti-tumor immune responses, thus the proportion of cytotoxic T cells (CTLs) in the spleen, TDLNs, and tumor tissues was also calculated via FCM analysis **(Figure 6B and Figure S20A)**. As expected, all treatments increased the CTL population in each tissue compared to the control. The combination treatment led to the most significant enhancement, with 2.22-fold, 2.13-fold, and 2.27-fold increases in CTL numbers in the spleen, TDLNs, and tumor tissues, respectively. This indicates that OCT@ES-mediated adjuvanticity, along with αPD-L1-mediated T-cell activation, strongly stimulated the expansion of cytotoxic T cells. The regulatory T cells are a major source of immune suppression in the tumor microenvironment, thus we conducted FCM to investigate whether the interventions could reverse this immunosuppressive effect 61. As revealed by **Figure 6C and Figure S20A**, the number of Tregs was reduced in all treatment groups. Notably, the proportion of immunosuppressive Tregs in the combinational treatment group was substantially lower than in the other groups, with nearly half the proportion of Tregs compared to the control group, both in the TDLNs and tumor tissues. This suggests that the combinational treatment was highly effective in reversing immunosuppression. Treg depletion was further confirmed by IF staining of tumor sections **Figure 6D and Figure S17**. Despite an increased proportion of CTLs, many CTLs in the tumor site may remain dysfunctional due to the immunosuppressive TME. To assess the cytolytic activity of CD^8+^ T cells, we analyzed the frequency of CD^8+^ T cells expressing key cytotoxic molecules (granzyme B, TNFα, perforin, and IFNγ) via intracellular FCM 54. As expected, OMV, αPD-L1, and OCT@ES treatments all induced a substantial increase in the expression of these cytotoxic molecules on CD8^+^ T cells compared to the control group **(Figure 6E and Figure S20B)**.

The combinational treatment led to the highest increase in cytotoxic molecule expression on CD8^+^ T cells, which is indicative of a more potent cytolytic response. Matured functional T cells often upregulate immune checkpoints, such as PD-1, to prevent excessive activation and maintain immune homeostasis. We also measured the proportion of PD-1^+^CD8^+^ T cells, as previous studies have shown that OMV treatment at the tumor site can increase PD-1 expression on CTLs 54. As expected, all treatments enhanced the expression of PD-1 on CD8^+^ T cells, likely due to the self-regulatory mechanism that prevents excessive CTL activation** (Figure 6E and Figure S20B)**. However, CTL becomes fully exhausted in the presence of PD-L1, a ligand that was frequently present on the tumor cell surface to achieve immune evasion and deplete CTL. These findings support the rationale for combining αPD-L1 with OCT@ES to maximize antitumor immunity. To assess whether the combinational therapy could establish long-term immune memory, we evaluated the proportion of memory T cells (Tem) at the tumor site after the final treatment. The results showed that the proportion of Tem was slightly elevated in all four treatment groups, indicating that long-term immune memory was not immediately established** (Figure S21)**. However, we observed that the proportion of granzyme B-positive CTLs was significantly increased in all treatment groups, with the combinational treatment showing the greatest increase—4.1-fold higher than the control group. Granzyme B is known to be a key effector molecule responsible for the tumoricidal activity of CTLs. Interestingly, granzyme B has also been linked to GSDME-dependent pyroptotic signaling in tumor cells 62, 63. Based on the previous observation that OCT@ES induced caspase-11-GSDMD-dependent pyroptosis through LPS sensing, we hypothesized that granzyme B might further amplify the intensity of pyroptosis through a GSDME-dependent pathway. To validate this hypothesis, we conducted IF staining of granzyme B and GSDME in tumor sections from different treatment groups. As expected, the combinational therapy group exhibited the highest expression of both granzyme B and GSDME, confirming that the combination of OCT@ES and αPD-L1 not only induced robust antitumor immunity but also amplified pyroptosis through granzyme B and GSDME signaling (**Figure 6F, Figure S18, and Figure S19**).

In addition to FCM analysis of immune cell populations, we measured the levels of key cytokines and immunogenic factors to further corroborate the changes in the tumor immune microenvironment **(Figure S20C)**. Serum levels of IL-6 and TNF-α were elevated in all treatment groups compared to the control, with the combination group showing the greatest increase, indicating effective DC maturation and immune activation. Intratumoral levels of IL-1β, LDH, ATP, and HMGB1 were also significantly higher in all treatment groups, with the combination group showing the highest expression, suggesting robust induction of pyroptosis and ICD.

In summary, the combination of OCT@ES and αPD-L1 effectively reprogrammed the immune-suppressive tumor microenvironment into an immune-activated state. This was achieved by inducing robust DC maturation, enhancing CTL activation, and reversing immunosuppression mediated by Tregs. Furthermore, the combination treatment amplified pyroptosis through granzyme B and GSDME signaling, providing a potent strategy for cancer immunotherapy.

### The long-term protective effect on tumor rechallenged models and lung metastasis models

**Figure 7:**
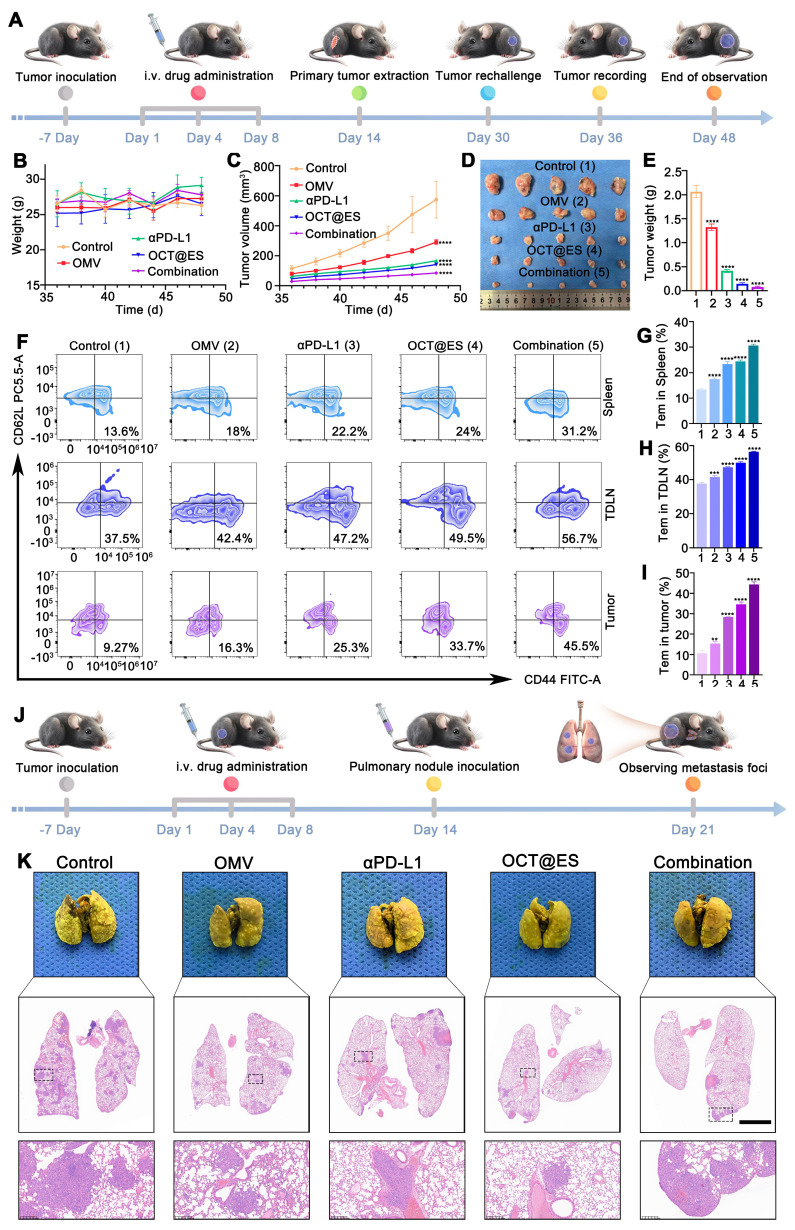
The long-term protective effect on tumor rechallenged models and lung metastasis models. (A) The schematic illustration of the establishment of tumor rechallenged models. (B) The average body weight change of the tumor rechallenged models after different treatments. Data are shown as the mean values ± SD (n = 5). (C) The tumor volume change of the tumor rechallenged models after different treatments. Data are shown as the mean values ± SD (n = 5). (D) The digital photograph of tumors extracted from the tumor rechallenged models at sacrifice on the 48^th^ day. (E) The weight of tumors extracted from the tumor rechallenged models at sacrifice on the 48^th^ day. Data are shown as the mean values ± SD (n = 5). (F) Representative FCM results of Tem (CD3^+^CD8^+^CD44^+^CD62L^+^) within spleens, TDLNs, and tumors after different treatments. (G, H, I) Quantitative analysis of Tem in spleens, TDLNs, or tumors, respectively. Data are shown as the mean values ± SD (n = 3). (J) The schematic illustration of the establishment of lung metastasis models. (K) The digital photos and H&E staining of lung tissues immersed by Bouin's fixative solution after different treatments. Scale bar: 2.5 mm. All the statistical significance was analyzed by ANOVA, ∗∗ *p <* 0.01, ∗∗∗ *p <* 0.001, ∗∗∗∗ *p <* 0.0001, compared with the control group.

Long-term protection plays an important role in preventing local tumor progression, distant metastases, and recurrence. Encouraged by the enhancement of T-cell protective immunity induced by the combinational therapy in hepa1-6 mouse models, we established tumor-rechallenged models and lung metastasis models to evaluate the long-term immune memory against tumor growth. The procedure for establishing the tumor-rechallenged model is depicted in** Figure 7A**. The body weight of all rechallenged models remained stable, demonstrating the biosafety of all treatments **(Figure 7B)**. In comparison to the control group, which exhibited rapid tumor growth, the rechallenged tumor on the left thigh of mice grew more slowly in all treatment groups. Among these, the combinational therapy group demonstrated the strongest tumor inhibition** (Figure 7C)**. Tumor images and weights extracted on day 48 confirmed these findings** (Figure 7D, and 7E)**. The tumor growth curve further illustrated the effective antitumor effect provided by long-term immune memory induced by OCT@ES and αPD-L1. Afterward, tumor tissues, TDLNs, and spleens were obtained and underwent FCM analysis to evaluate the proportion of Tem (CD3^+^CD8^+^CD44^+^CD62L^+^) which are derived from CTL and play a key role in long-lasting antitumor immune memory **(Figure 7F, 7G, 7H, and 7I)**. FCM analysis revealed that Tem populations were significantly increased in mice treated with the combinational therapy compared to those in other groups. Specifically, in the spleen and TDLNs, the proportion of Tem was slightly higher in the OMV, αPD-L1, or OCT@ES-treated groups, while the combination group showed a 2.3-fold increase in the spleen and 1.5-fold increase in TDLNs compared to the control. Notably, the proportion of Tem was significantly higher in the secondary tumors. Compared to the control group, the number of Tem in the OMV, αPD-L1, and OCT@ES-treated groups increased by 1.76-fold, 2.73-fold, and 3.64-fold, respectively. The combination treatment resulted in a 4.91-fold increase in Tem, demonstrating the potent antitumor immune memory induced by αPD-L1 and OCT@ES. This vigorous immune memory offers effective protection against tumor recurrence.

To evaluate whether the combinational therapy could also suppress tumor metastasis, we established lung metastasis models by injecting Hepa1-6 tumor cells into mice after different treatments via the tail vein** (Figure 7J)**. One week after tumor cell injection, the lung tissues of all mice were collected, photographed, and subjected to H&E staining to assess lung metastasis. As shown in the H&E staining images, the control group exhibited a high degree of canceration, with numerous metastatic nodules distributed throughout the lung tissue. In contrast, the other treatment groups showed significantly reduced metastatic nodule areas, indicating effective suppression of metastasis. This suggests that the combinational therapy—through robust activation of the antitumor immune response—was highly effective in inhibiting tumor metastasis **(Figure 7K and Figure S22)**.

### Evaluation of biosafety *in vivo*

To evaluate the biological safety of OCT@ES, 6-8-week-old C57BL/6J mice were treated with *i.v.* injections of OCT@ES on days 0, 1, 3, 7, 14, and 28. On the 28^th^ day, all mice were sacrificed, and whole blood, blood serum, and major organs were collected for blood biochemistry analysis and histological examination. **Figure S23A and Figure S23B** demonstrated that the administration of OCT@ES did not cause significant changes in the major hematology and biochemical parameters, indicating that OCT@ES did not induce harmful effects on hematological, liver, or kidney functions at the doses used in the *in vivo* treatment. As demonstrated by **Figure S23C**, the H&E staining of major organs exhibited no obvious inflammation and morphological abnormalities, implying no pathological toxicities and adverse events. Overall, the results indicate that OCT@ES is biocompatible and poses no significant threat to major organs, making it a promising candidate for future clinical applications.

## Conclusion

In this study, we successfully developed a sophisticated CPApoptosis nano-actuator (OCT@ES) using a nano-immuno-engineering approach. OCT@ES was shown to efficiently accumulate at the tumor site, enter tumor cells via endocytosis, and release its components in response to acidic conditions, resulting in a potent antitumor effect in both Hepa1-6 and CT26 mouse models. This was achieved through the activation of multiple cell death pathways, including pyroptosis, cuproptosis, and apoptosis, while simultaneously triggering an immunological switch. The intensive immunogenic cell death (ICD) induced by “CPApoptosis” effectively stimulated both the innate and adaptive immune systems, as evidenced by the upregulation of matured dendritic cells (DCs) and an increased proportion of CTLs. The combination of OCT@ES with αPD-L1 further amplified the antitumor immunity and long-term protection, as demonstrated in the rechallenged tumor models. Notably, granzyme B secreted by CTLs activated GSDME-dependent pyroptosis, creating a tumor-killing loop. Overall, this study introduces a multi-pathway cell death strategy that targets tumor progression, recurrence, and metastasis. However, several improvements should be considered to maximize therapeutic efficacy, including enhancing the targeting performance of OCT@ES, optimizing the dose-effect relationship among Cu²⁺, TA, ES, and αPD-L1, exploring MR-enabled theranostic applications, and evaluating the broader impact of OCT@ES on the immune landscape beyond T cells.

## Supplementary Material

Supplementary figures.

## Abbreviations

ICD: Immunogenic cell death
ROS: Reactive oxygen species
CRT: Calreticulin
DAMPs: Damage-associated molecular patterns
TAAs: tumor-associated antigens
OMV: outer membrane vesicle
DLAT: Dihydrolipoamide acetyltransferase
Fe-S: Iron-sulfur
DC: dentritic cell
GSH: Glutathione
ES: Elesclomol
TA: Tannic acid
MPNs: Metal-phenolic networks
MPS: Mononuclear phagocytic system
EPR: Enhanced permeability retention
ELISAs: Enzyme-linked immunosorbent assays
TEM: Transmission emission microscopy
ICP-MS: Inductively coupled plasma-mass spectrometry
XPS: X-ray photoelectron spectroscopy
XRD: X-ray diffractometer
VSM: Vibrating Sample Magnetometer
FTIR: Fourier transform infrared spectrometer
HPLC: High performance liquid chromatography
CLSM: Confocal laser scanning microscope
FCM: Flow cytometry
TUNEL: Terminal deoxynucleotidyl transferase dUTP nick end labeling
PCNA: Proliferating cell nuclear antigen
H&E: hematoxylin and eosin
DHE: Dihydroethidium
FL: fluorescence
TDLNs: tumor draining lymph nodes
ANOVA: One unpaired multiple *t*-test and analysis of variance
DLS: Dynamic light scattering
CTLs: Cytotoxic T cells
Tem: Memory T cells
