# Supplementary material for: A “CPApoptosis” nano-actuator switches immune-off solid tumors to immune-on for fueling T-cell- based immunotherapy
Source: Theranostics. 2025 Mar 3;15(9):3797–820. doi: 10.7150/thno.105867 (PMC11980655; doi:10.7150/thno.105867)
Supplement: Supplementary file 1 — Supplementary figures. [file thnov15p3797s1.pdf]

Supporting information

A "CPApoptosis" nano-actuator switches immune-off solid tumors to immune-on for fueling T-cell- based immunotherapy

*Ying Luo[1,2*], Yi Wang[1*], Bo Liu[1], Yun Liu[1], Wenli Zhang[1], Sijin Chen[1], Xiyue Rong[1], Lian Xu[1], Qianying Du[1], Jia Liu[1], Jie Xu[1], Haitao Ran[2], Zhigang Wang[2], Dajing Guo[1]*

Department of Radiology, Second Affiliated Hospital of Chongqing Medical University, Chongqing, 400010, P.R. China

Chongqing Key Laboratory of Ultrasound Molecular Imaging & Department of Ultrasound, Second Affiliated Hospital of Chongqing Medical University, Chongqing, 400010, PR China

*Corresponding author: guodaj@hospital.cqmu.edu.cn (Dajing Guo)

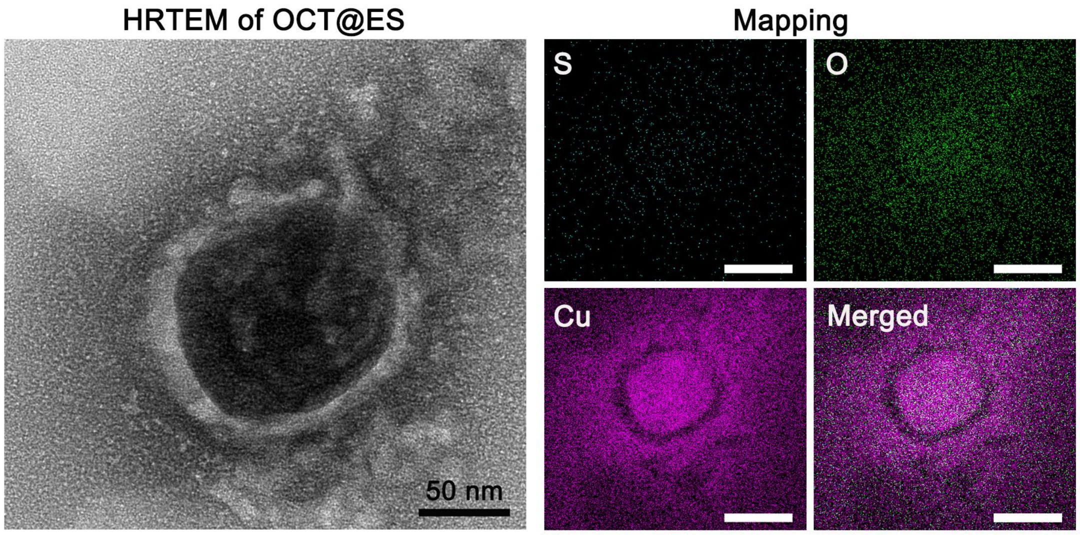


Figure S1. The high resolution TEM of OCT@ES and TEM mapping. Scale bar: 50 nm.

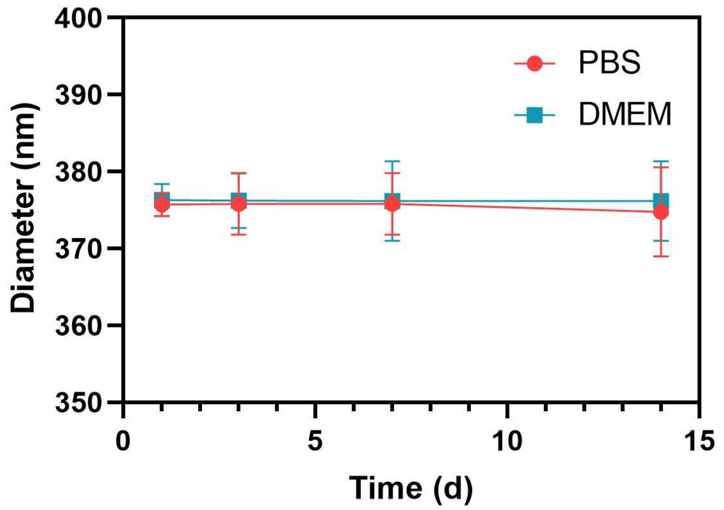


Figure S2 . The stability of OCT@ES in both PBS and DMEM medium as detected by DLS.

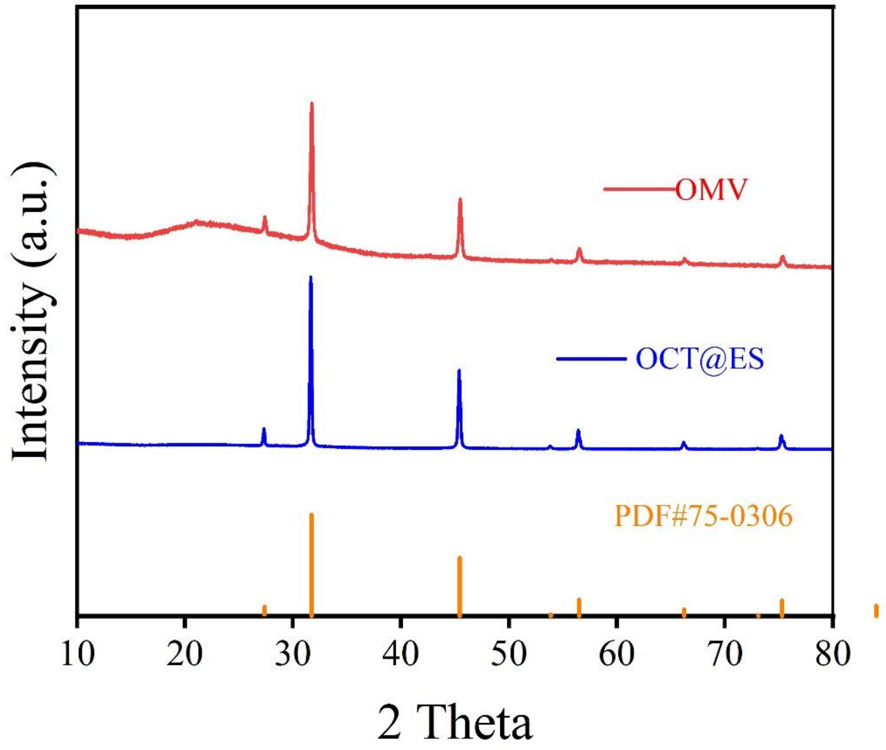


Figure S3. The XRD patterns of OMV and OCT@ES.

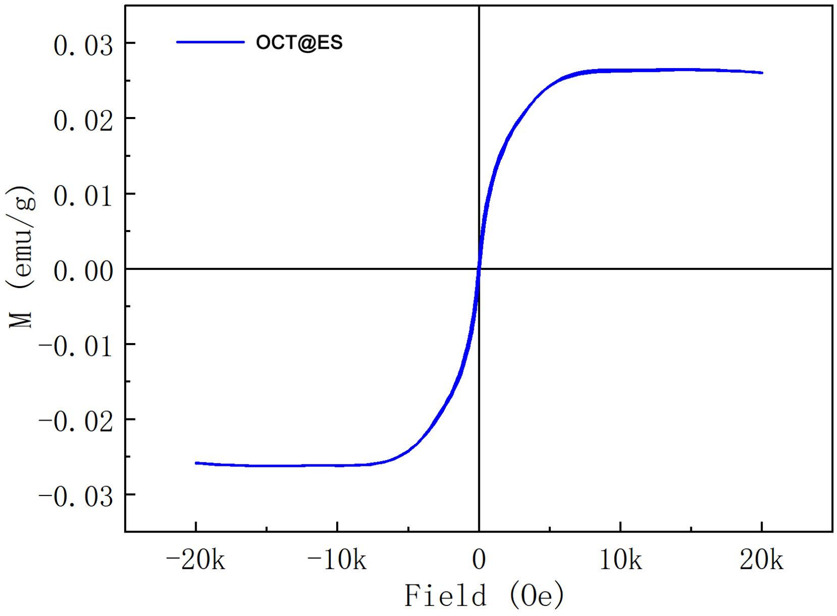

Figure S4. The hysteresis loop of OCT@ES as detected by VSM.

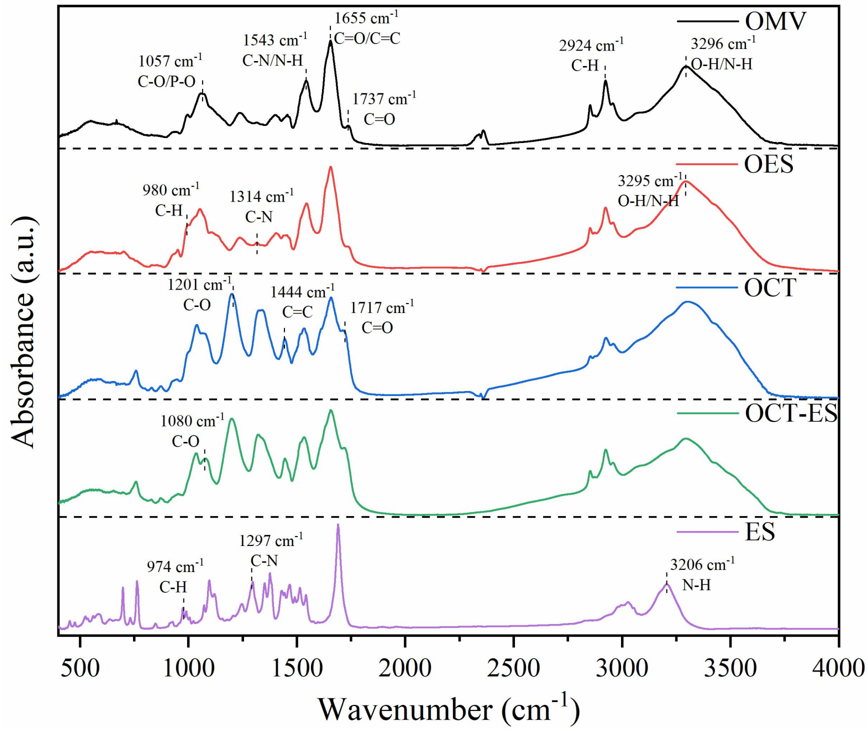

Figure S5. The FTIR spectra of OMV, OES, OCT, OCT@ES, and ES, respectively.

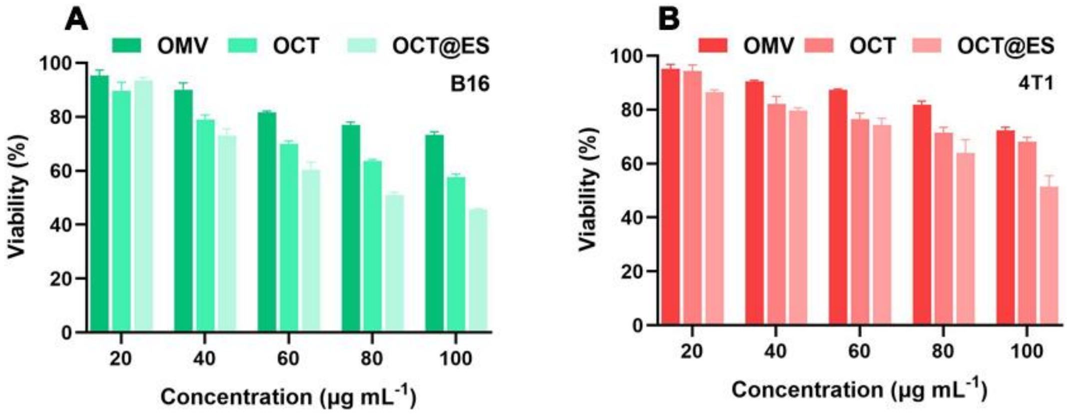


Figure S6. The cytotoxicity of OMV, OCT, and OCT@ES on (A) B16 and (B) 4T1 mouse tumor cells. Data are shown as the mean values ± SD (n = 3).

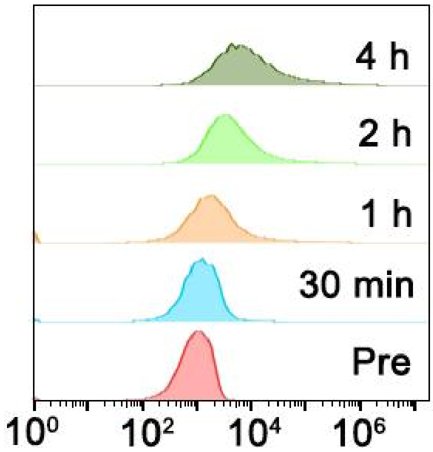


Figure S7. The FCM analysis of intracellular uptake of DiI-labeled OCT@ES by hepa1-6 cells after different co-incubation time.

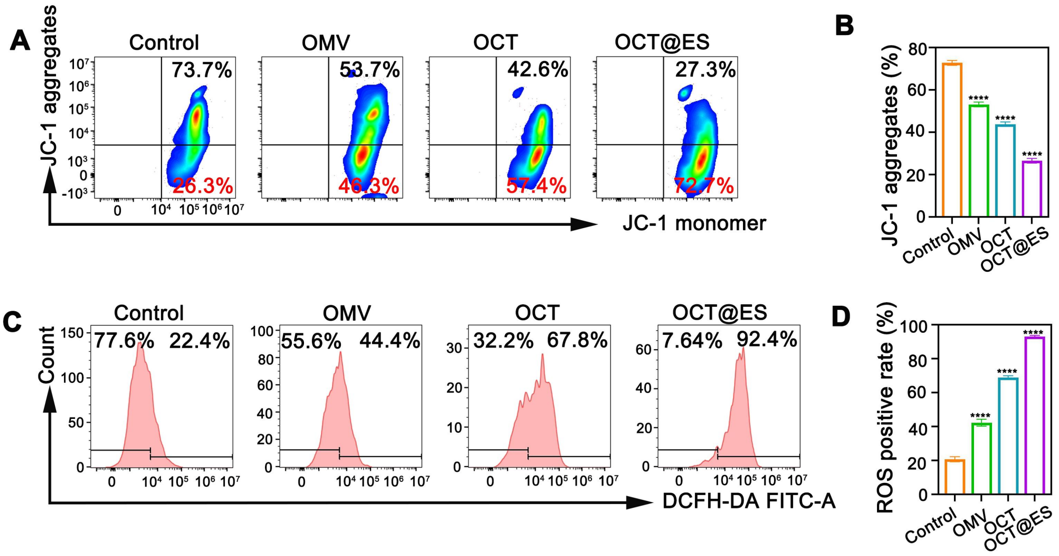


Figure S8. (A, B) The FCM analysis of JC-1 in different treatment groups and the quantitative analysis of JC-1 aggregates. Data are shown as the mean values ± SD (n = 3). (C, D) FCM analysis of DCFH-DA expression in different treatment groups. Data are shown as the mean values ± SD (n = 3). All the statistical significance was analyzed by ANOVA, ∗∗∗∗ $p < 0.0001$, compared with the control group.

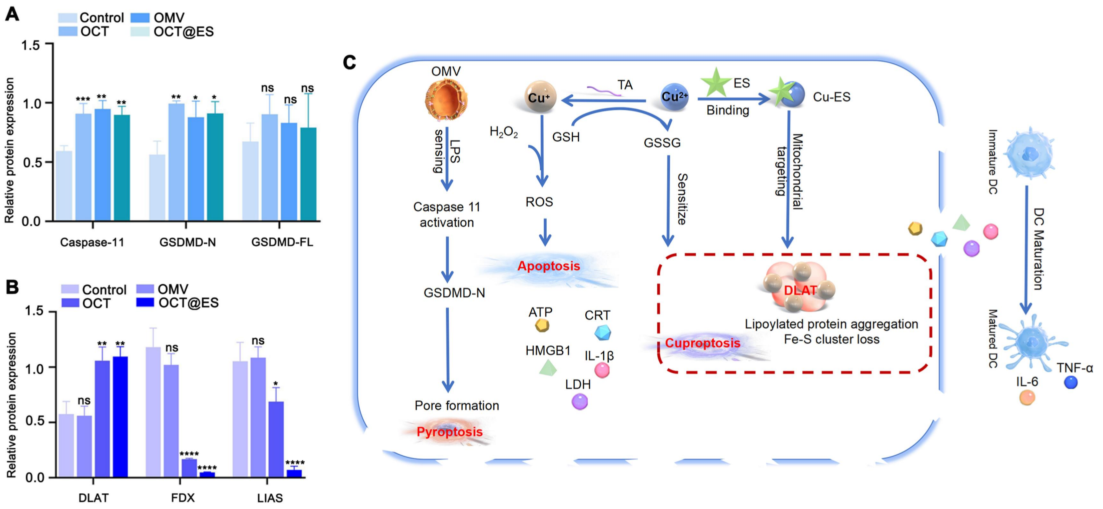


Figure S9. (A) The quantitative analysis of caspase-11, GSDMD-N, and GSDMD-FL expression in different treatment groups. Data are shown as the mean values ± SD (n = 3). (B) The quantitative analysis of DLAT, FDX, LIAS expression in different treatment groups. Data are shown as the mean values ± SD (n = 3). (C) The illustrative mechanism of OCT@ES-induced CPApoptosis. All the statistical significance was analyzed by ANOVA, ∗ $p < 0.05$, ∗∗ $p < 0.01$, ∗∗∗ $p < 0.001$, ∗∗∗∗ $p < 0.0001$, ns, not significant, compared with the control group.

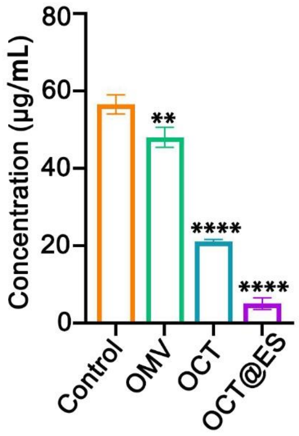


Figure S10. The intracellular GSH assay in different treatment groups. Data are shown as the mean values ± SD (n = 3). All the statistical significance was analyzed by ANOVA, ＊＊ $p < 0.01$, ＊＊＊＊ $p < 0.0001$, compared with the control group.

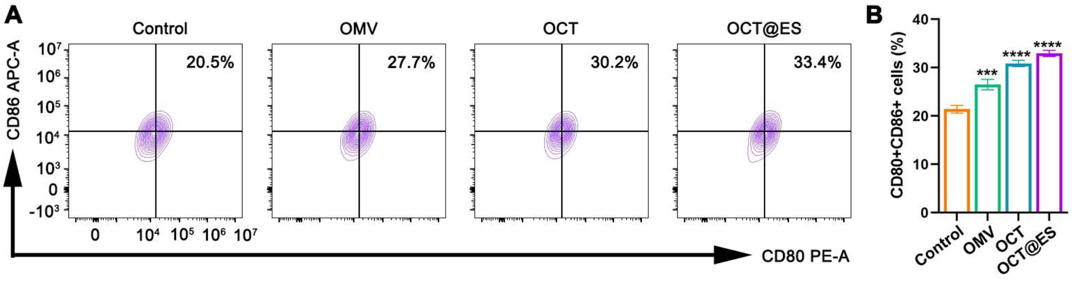


Figure S11. (A) The FCM analysis of the effect of mere OMV, OCT, and OCT@ES on JAWSII cells through FCM and (B) statistical analysis. Data are shown as the mean values ± SD (n = 3). All the statistical significance was analyzed by ANOVA. ＊ $p < 0.05$, ＊＊ $p < 0.01$, ＊＊＊ $p < 0.001$, ＊＊＊＊ $p < 0.0001$, ns, not significant.

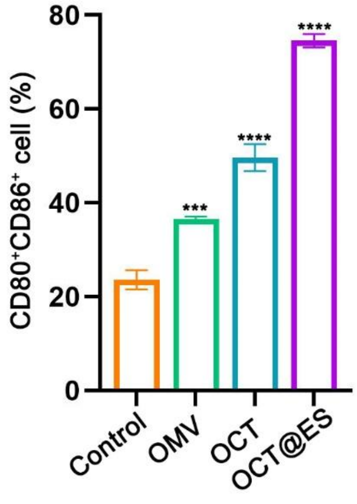


Figure S12. (a) The FCM proportion of $CD11c^{+}CD80^{+}CD86^{+}$ cells in different treatment groups. Data are shown as the mean values ± SD (n = 3). All the statistical significance was analyzed by

ANOVA, * * * $p < 0.001$, * * * * $p < 0.0001$, compared with the control group.

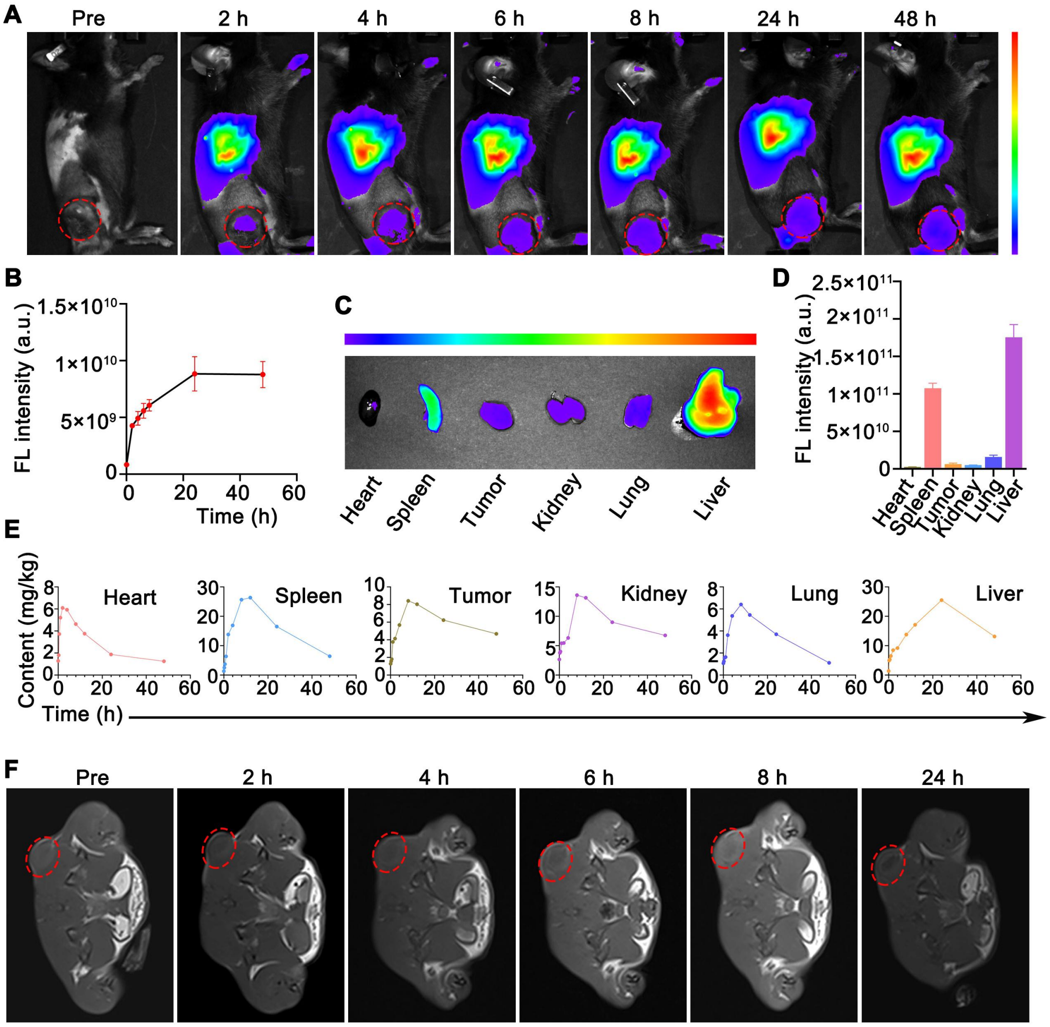


Figure S13. (A, B) In vivo FL images of mice captured at different time intervals after the injection of DiR-labeled OCT@ES and the corresponding quantitative FL analysis. Data are shown as the mean values ± SD (n = 3). (C, D) The ex vivo FL images of the extracted tumors and major organs (Spleen, heart, liver, lung, kidney) and the corresponding quantitative FL analysis. Data are shown as the mean values ± SD (n = 3). (E) The in vivo Cu pharmaceutical kinetics in tumor and major organs (spleen, liver, heart, kidney, and lung). (F) Representative MR gray image of the tumor-bearing mice at different time points after the i.v. injection of OCT@ES.

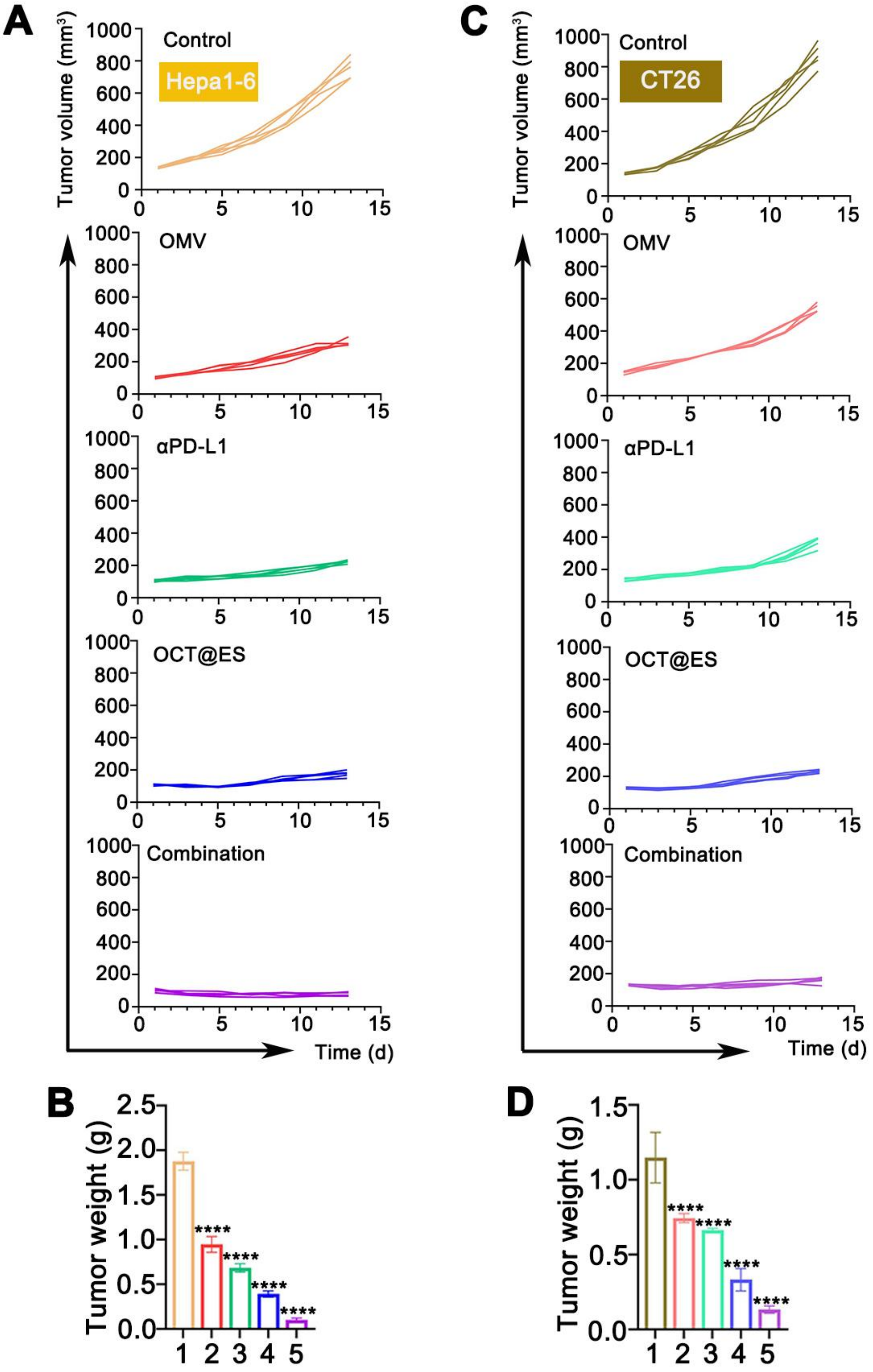


Figure S14. (A) The individual tumor growth curve of the hepa1-6 mouse model in each treatment group. Data are shown as the mean values ± SD (n = 5). (B) The weight of tumors extracted from the hepa1-6 mouse models in different groups. Data are shown as the mean values ± SD (n = 5).

(C) The individual tumor growth curve of the CT26 mouse model in each treatment group. Data are shown as the mean values ± SD (n = 5). (D) The weight of tumors extracted from the CT26 mouse models in different groups. Data are shown as the mean values ± SD (n = 5). All the statistical significance was analyzed by ANOVA, **** $p < 0.0001$, compared with the control group.

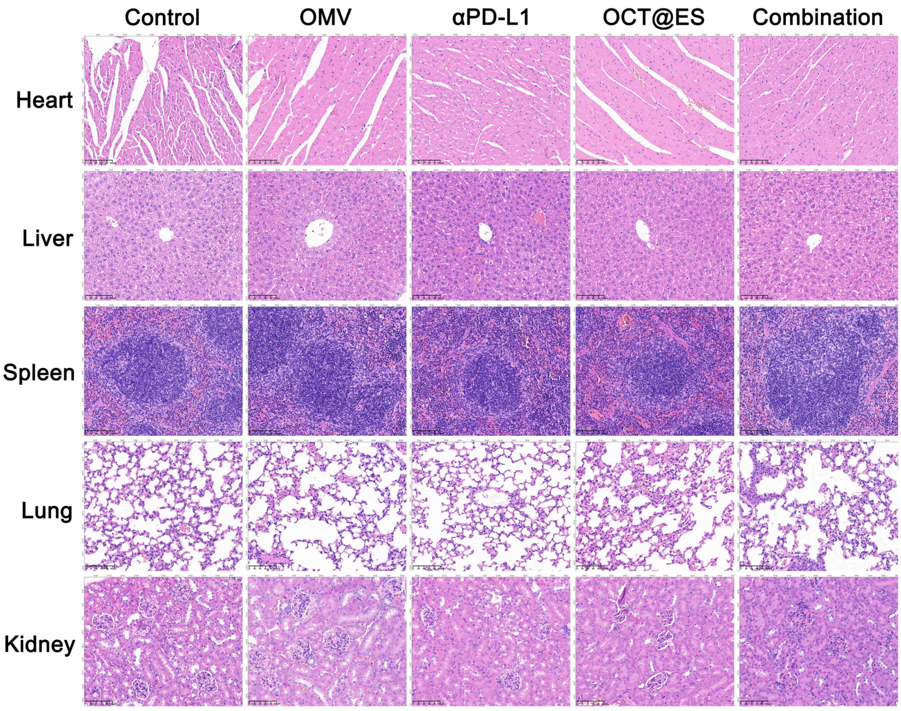


Figure S15. H&E staining of the major organs collected from mice after different treatments. Scale bar: 100 μm.

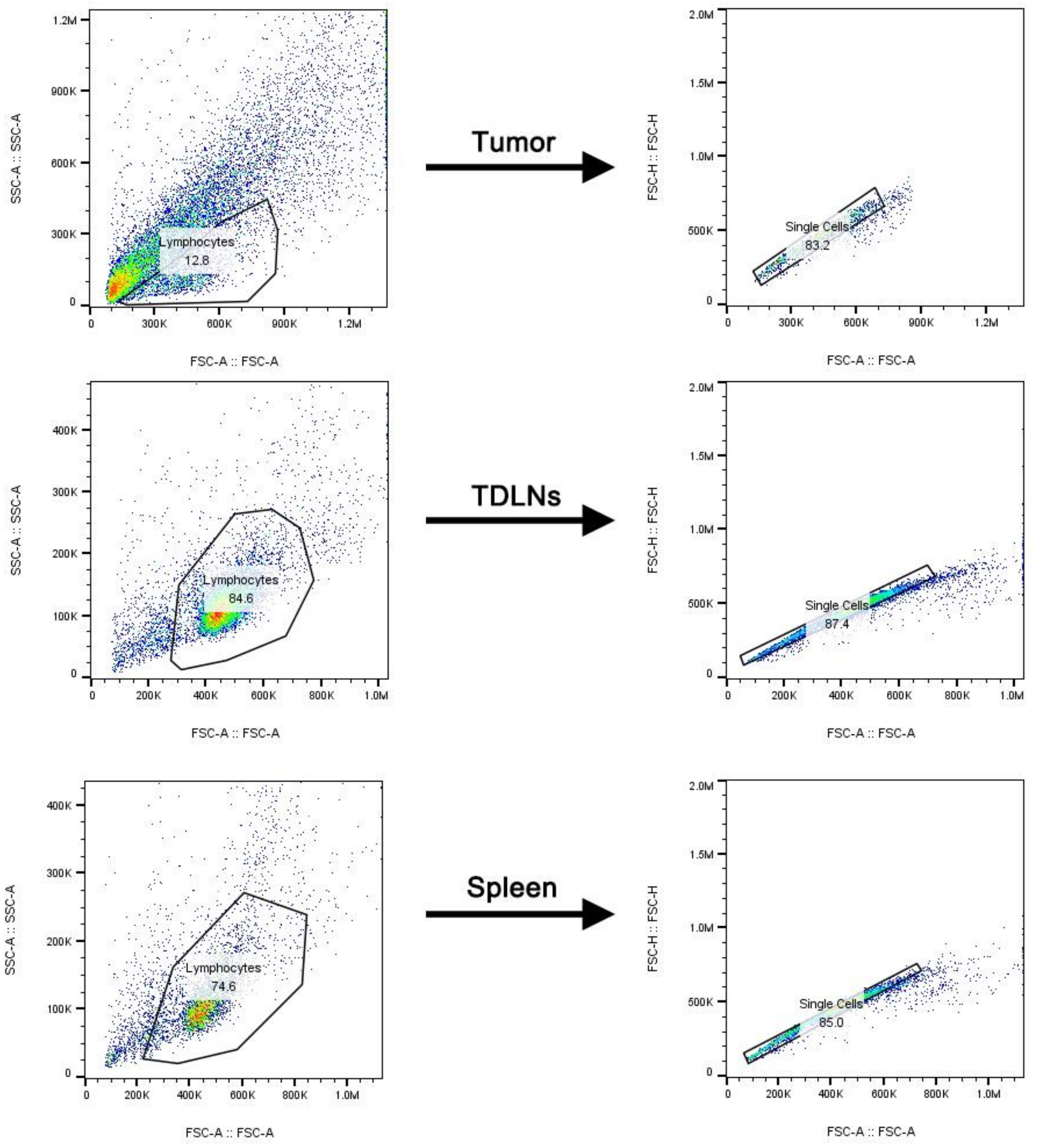


Figure S16. Gating plots (FSC-A/SSC-A and FSC-A/FSC-H) of lymphocyte analysis in the tumor sites , TDLNs, and spleens in Figure 6 and Figure 7.

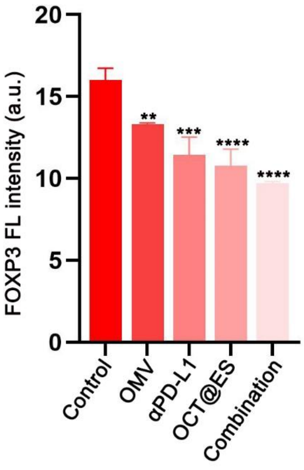

Figure S17. The FL quantitative analysis of the FOXP3 expression in tumor sections from different treatment groups. Data are shown as the mean values ± SD (n = 3). All the statistical significance was analyzed by ANOVA, ** $p < 0.01$, *** $p < 0.001$, **** $p < 0.0001$, compared with the control group.

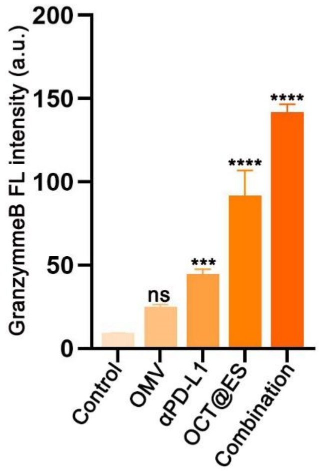


Figure S18. The FL quantitative analysis of the granzyme B expression in tumor sections from different treatment groups. Data are shown as the mean values ± SD (n = 3). All the statistical significance was analyzed by ANOVA, *** $p < 0.001$, **** $p < 0.0001$, ns, not significant, compared with the control group.

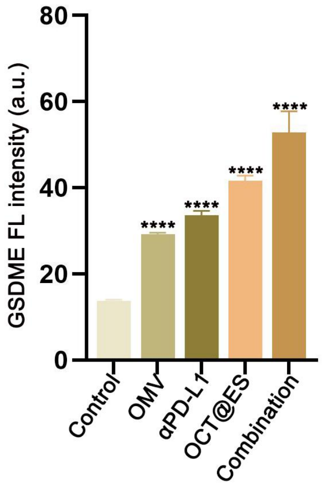


Figure S19. The FL quantitative analysis of the GSDME expression in tumor sections from different treatment groups. Data are shown as the mean values ± SD (n = 3). All the statistical significance was analyzed by ANOVA, **** $p < 0.0001$, compared with the control group.

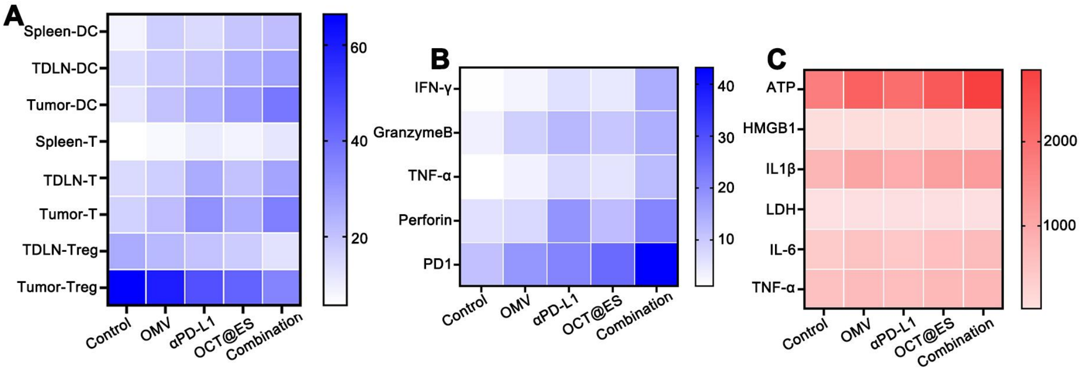


Figure S20. (A) The statistical analysis of immune cells within the spleens, TDLNs, and tumors after different treatments. Data are shown as the mean values ± SD (n = 3). (B) The statistical analysis of cytolytic T-cell expression within the tumors after different treatments. Data are shown as the mean values ± SD (n = 3). (C) The ELISA test of ATP, HMGB1, IL1β, LDH, IL-6, and TNF-α. Data are shown as the mean values ± SD (n = 3).

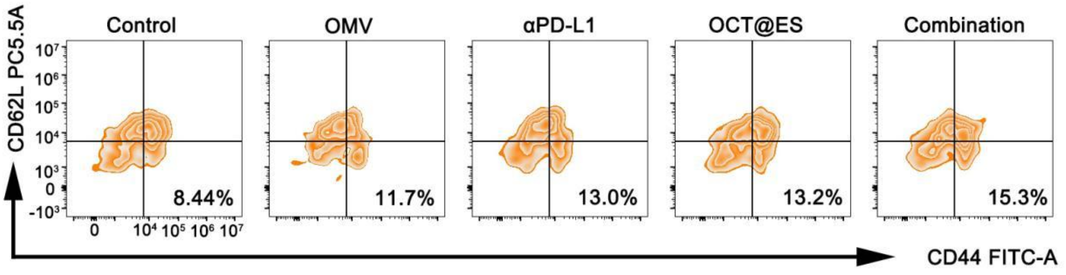


Figure S21. The FCM analysis of $T_{em}$ after treatment.

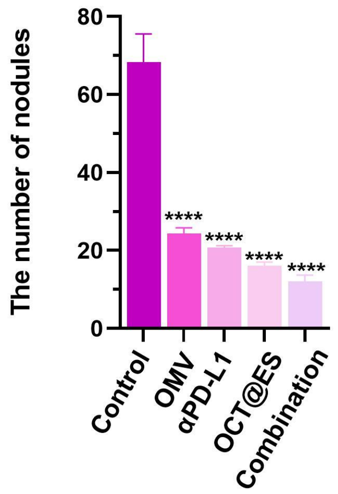


Figure S22. statistical analysis on the number of lung metastasis nodules in different groups in H&E staining. Data are shown as the mean values ± SD (n = 3). All the statistical significance was analyzed by ANOVA, ＊＊＊＊ $p < 0.0001$, compared with the control group.

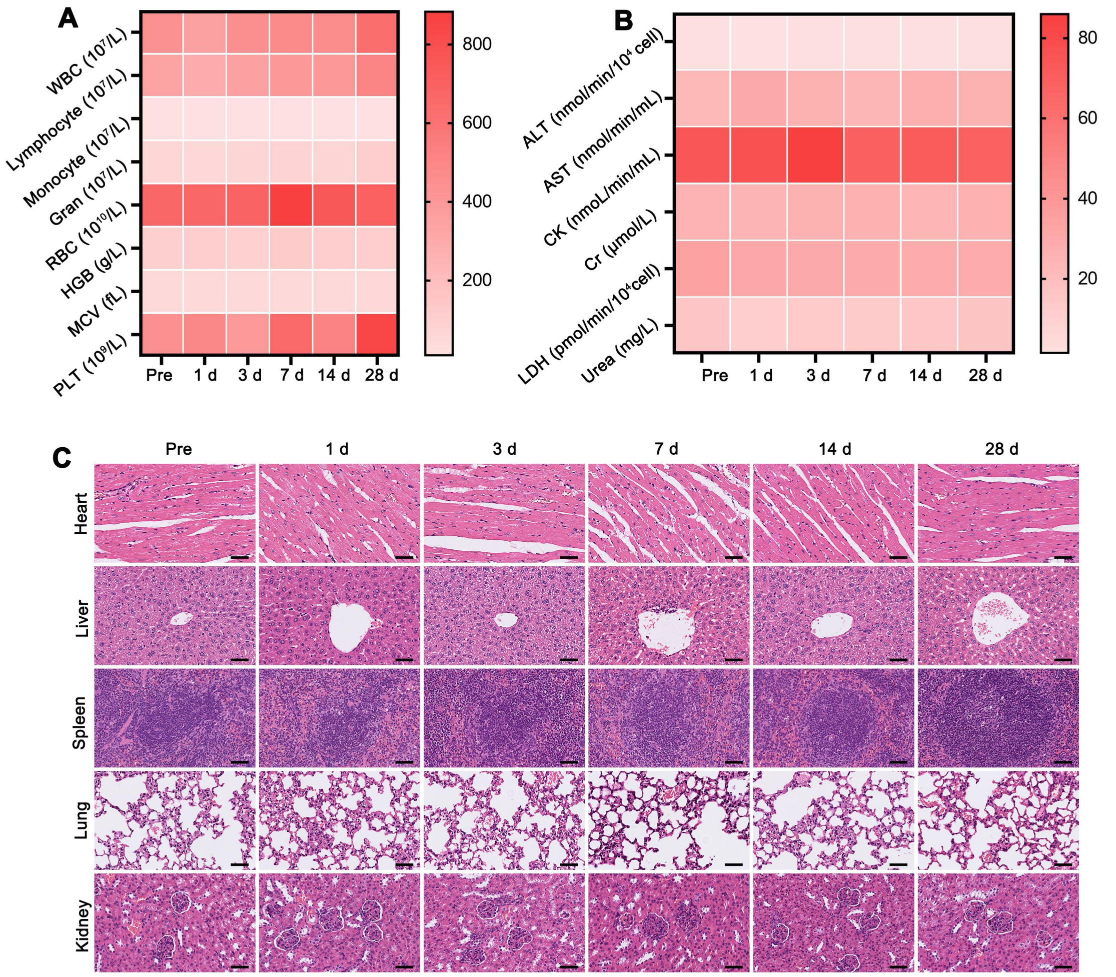


Figure S23. (A) Blood biochemical parameters and (B) blood routine examination of mice 21 days after intravenous injection of OPCM. Data are shown as the mean values ± SD (n = 3). (C) H&E staining of the major organs at corresponding time intervals. Scale bar: 50 μm.
